# Transcriptomic profiling of an evolved *Yarrowia lipolytica* strain: tackling hexanoic acid fermentation to increase lipid production from short-chain fatty acids

**DOI:** 10.1186/s12934-024-02367-4

**Published:** 2024-04-03

**Authors:** Sergio Morales-Palomo, Clara Navarrete, José Luis Martínez, Cristina González-Fernández, Elia Tomás-Pejó

**Affiliations:** 1 Biotechnological Processes Unit, IMDEA Energy, Móstoles (Madrid), Spain; 2 Department of Chemical Engineering and Environmental Technology, School of Industrial Engineering, Valladolid University, Valladolid, 47011 Spain; 3 Institute of Sustainable Processes, Valladolid, 47011 Spain; 4 Department of Biotechnology and Biomedicine, Technical University of Denmark, Søltofts Plads Building 223, Kgs. Lyngby, 2800 Denmark

**Keywords:** Adaptive laboratory evolution, Microbial oils, Yeast lipids, *Yarrowia lipolytica*, Hexanoic acid

## Abstract

**Background:**

Short-chain fatty acids (SCFAs) are cost-effective carbon sources for an affordable production of lipids. Hexanoic acid, the acid with the longest carbon chain in the SCFAs pool, is produced in anaerobic fermentation of organic residues and its use is very challenging, even inhibiting oleaginous yeasts growth.

**Results:**

In this investigation, an adaptive laboratory evolution (ALE) was performed to improve *Yarrowia lipolytica* ACA DC 50109 tolerance to high hexanoic acid concentrations. Following ALE, the transcriptomic analysis revealed several genetic adaptations that improved the assimilation of this carbon source in the evolved strain compared to the wild type (WT). Indeed, the evolved strain presented a high expression of the up-regulated gene YALI0 E16016g, which codes for FAT1 and is related to lipid droplets formation and responsible for mobilizing long-chain acids within the cell. Strikingly, acetic acid and other carbohydrate transporters were over-expressed in the WT strain.

**Conclusions:**

A more tolerant yeast strain able to attain higher lipid content under the presence of high concentrations of hexanoic acid has been obtained. Results provided novel information regarding the assimilation of hexanoic acid in yeasts.

**Supplementary Information:**

The online version contains supplementary material available at 10.1186/s12934-024-02367-4.

## Background

Vegetable oils cannot meet the increasing demand for oleochemical production required in different industrial sectors (e.g., biofuels, lubricants, cosmetics, etc.). To broaden the oil resources available to the chemical industry, microbial oils have emerged as an interesting alternative. These oils are intracellularly produced by some microorganisms. Among all oleaginous microorganisms, yeasts are particularly promising due to their ability to accumulate lipids up to 50% w/w of their dry weight, their high growth rates, short life cycles and carbon sources versatility [1].

*Yarrowia lipolytica* has been extensively used in the last few years to produce lipids using sugars, such as glucose and xylose [2–5]. *Y. lipolytica* has emerged as a novel microbial chassis for metabolic engineering [6] and its genetic background has been thoroughly examined, leading to the publication of different genome sequences for several *Y. lipolytica* strains [7]. Most operational processes and advanced genetic engineering tools have been designed to optimize lipid production in *Y. lipolytica* via sugar’s assimilation pathways [8]. Nevertheless, the price of using sugars as carbon source can account for 60–80% of the total production cost [9]. Due to the unique metabolic characteristics of *Y. lipolytica*, the yeast is able to assimilate non-sugar substrates derived from organic residues, such as short-chain fatty acids (SCFAs) [10, 11]. SCFAs are organic acids with great potential as low-cost carbon source for lipid production [12] and can be produced through the anaerobic fermentation of organic wastes [13]. However, the use of these SCFAs as carbon source is challenging because they can inhibit yeast growth [14].

Recent studies have pointed out that inhibitory effects associated with SCFAs in yeasts do not only lie in the total concentration of acids but in the SCFAs distribution profile [10]. In the anaerobic fermentation process of certain wastes, acetic and hexanoic acids are present in the resulting digestates [15]. Hexanoic acid, the fatty acid with the longest chain among the SCFAs, is the most inhibitory and the most difficult to assimilate for yeast [13]. Because of that, it would be tremendously beneficial to have a yeast capable of efficiently use hexanoic acid-rich digestates to make the industrial process suitable for a circular economy. So far, only the metabolic assimilation pathways of acetic and propionic acid, the shortest SCFAs, have been described [16]. Therefore, the genetic engineering modifications necessary to promote hexanoic acid assimilation whilst mitigating its inhibitory effect are still unresolved.

Adaptive Laboratory Evolution (ALE) has been previously used to obtain yeast mutants with enhanced performances. This approach is an effective strategy to increase yeasts robustness both in WT and in engineered strains [17]. Beneficial phenotypes appear through the accumulation of spontaneous mutations across successive generations under constant selection pressure [18]. This methodology allows the development of improved microbial strains without the necessity for an intricate understanding of inhibitory mechanisms or complex interactions between inhibitors and biochemical or genetic networks [19]. Additionally, ALE circumvents the metabolic burden associated with heterologous gene/protein expression and eliminates the requirement for strict control of genetic expression, which is typically needed in rational approaches [20]. Despite these advantages, few studies have conducted ALE using *Y. lipolytica* [21, 22]. Therefore, information on how this yeast behaves during ALE remains scarce.

To fill the gap of knowledge on yeast SCFAs metabolic assimilation and improve the use of the challenging SCFAs, *Y. lipolytica* ACA DC 50109 was subjected to ALE in the presence of high hexanoic acid concentration. Subsequently, a transcriptomic analysis of the evolved and wild type (WT) strains genomes was conducted to unveil some of the specific genes involved in the cellular processes for SCFAs metabolism.

## Materials and methods

### Yeast strain and preinoculum preparation

*Y. lipolytica* ACA DC 50109 (from the Agricultural University of Athens’ culture collection) was the oleaginous yeast used in this study. The yeast was preserved as described in Morales-Palomo et al. (2022) [23]. For preinoculum preparation, one colony of the strain was inoculated in YPD liquid medium (20 g/L peptone, 20 g/L glucose and 10 g/L yeast extract), with an initial pH of 7.0. The yeast cells were incubated overnight at 150 rpm and 27 ºC in a rotary shaker until the late exponential growth phase was reached.

### Fermentation media

The composition of the synthetic media (SM) used in this study is specified in Table 1. The chemicals used in the preparation of the SM exhibited a purity level of ≥ 99.9%.

**Table 1:** Composition of different synthetic media used in this study

	Concentration (g/L)			
	SM 1 (1:1)*	SM 2 (1:2)*	SM 3 (1:4)*	SM 4 (1:6)
Acetic acid	3.57	2.60	1.48	1.02
Propionic acid	0.64	0.61	0.68	0.66
Butyric acid	5.93	5.47	5.78	5.84
Valeric acid	1.42	1.33	1.31	1.29
Hexanoic acid	3.52	5.19	5.90	6.24
Total	15.08	15.20	15.15	15.06

* Acetic acid:hexanoic acid ratio (A:H) are in brackets

SM 1 was prepared according to the SCFAs profile obtained by Greses et al., (2020) in the anaerobic fermentation of food wastes [15]. To carry out the ALE of *Y. lipolytica*, SM 2 and 3 were modified from SM 1 to have higher hexanoic acid concentrations. In order to maintain 15 g/L of SCFAs in all media, the concentration of acetic acid, the least inhibitory acid, was reduced while hexanoic acid was increased. Thus, four different A:H ratios (1:1, 1:2, 1:4 and 1:6) were established, corresponding to SM 1, 2, 3 and 4. Since the YNB composition was the same in all media (1.7 g/L of yeast nitrogen base and 7.5 g/L of ammonium sulfate), the initial C:N ratio was kept at 3.5.

### Adaptive laboratory evolution of *Y. lipolytica*

The initial batch of ALE started by inoculating yeast cells from overnight YPD cultures to flasks containing 100 mL of SM 1 to achieve an initial OD of 0.8 at 600 nm (OD_600_) (≈ 0.4 g dry weight cells/L). ALE was performed in triplicates in baffled Erlenmeyer flasks of 250 mL. A working volume of 100 mL of SM at pH 6.0 was used. Shake flasks were incubated at 170 rpm and 27 ºC in a rotary shaker until 95–100% of the SCFAs were consumed. At this time point, an aliquot of cells was transferred in fresh 100 mL of SM with the same A:H at OD_600_ 0.8. The serial transfers of cells continued for adaptation in the given A:H ratio. When a significant improvement in terms of shorter lag phase was observed (faster SCFAs consumption and higher cell growth), cells were transferred to SM with an increased concentration of hexanoic acid. The A:H ratio increased gradually during repetitive batch cultures as evolution proceeded, being: 1:1, 1:2, 1:4 and 1:6.

In each round of adaptation (48–120 h), the population growth was equal to 5 doubling times. ALE was finished after a total of 27 adaptation rounds. This implies 135 generations for the whole ALE procedure. The lag phase, SCFAs consumption and growth rate were monitored throughout the entire ALE in each batch cultivation.

### Fermentation experiments: clone selection and WT vs. evolved strain comparison

During ALE, no selection of adapted cells in each round of adaptation was carried out, resulting in the transfer of both WT and adapted cells. However, under these restrictive conditions, the fittest cells were predominant. The most adapted cells to media with A:H ratio of 1:4 were isolated from the rest of cells in the population using the replica plating technique after 27 adaptation rounds. For this, three different plates with solid YNB (same concentration as previously described) with an A:H ratio of 1:1, 1:2 and 1:4 were prepared. Those colonies that exhibited growth at the same spot on the three plates were selected as isolated clones and preserved in 20% v/v glycerol at − 80 °C. A total of 6 isolated clones were used in a fermentation experiment of SM 3. The clone that showed the shortest lag phase, the highest SCFAs consumption and the highest growth rates was selected as the evolved strain (data not shown). The WT strain was also used in the fermentation of SM 3 for comparative purposes. Preinocula were prepared as described above (Sect. 2.1). Fermentations were inoculated with an initial OD of 0.8 and were incubated in a rotary shaker using the same parameters described in Sect. 2.3 until 95–100% of the SCFAs were consumed. At the end of the exponential phase, samples were collected for the quantification of the lipid content in both evolved and WT strains.

Samples for RNA sequencing were withdrawn at the beginning and end of the exponential phase of the evolved (40 h and 75 h, respectively) and the WT strain (70 h and 110 h, respectively) in fermentation tests of SM 3.

### Analytical methods

SCFAs quantification was conducted using high-pressure liquid chromatography (HPLC), following the methodology outlined in Morales-Palomo et al. (2022) [23]. The mobile phase consisted of 5 mM H_2_SO_4_ eluted in isocratic mode with a flow rate of 0.6 mL/min. The detector and oven temperatures were maintained at 35ºC and 50ºC, respectively.

Cell growth was followed at OD_600_ using a Spectroquant® Pharo 100 spectrophotometer. Dry biomass was determined by applying a pre-established standard curve that correlates the OD of the culture with yeast biomass production (g/L). Total nitrogen content in fermentation media was measured with a Nitrogen (total) Cell Test from Spectroquant® (Merck).

The quantification of lipid content was performed through fluorometric analysis using the optimized protocol described by Morales-Palomo et al. (2022) [23]. The area of each fluorescence curve was processed and converted to quantum yield.

### Gene expression analysis

#### RNA-seq data analysis

RNA library preparation and sequencing were performed by Novogen (https://www.novogene.com/), using an Illumina PE150 instrument (Illumina, San Diego, CA, USA).

The RNA-seq raw data was subjected to analysis and visualization using Galaxy (https://usegalaxy.org/), an open-source web-based platform for data integration and analysis. FastQC tool (Babraham Bioinformatics) was used for quality control of the raw data generated from the sequencing pipelines. Afterward, results were visualized using MultiQC [24].

The alignment of RNA-seq data to *Y. lipolytica* ACA DC 50109 reference genome (obtained from the Pubmed genome database (https://pubmed.ncbi.nlm.nih.gov/); *Y. lipolytica* CLIB122, NCBI access number: ASM 252v1) was performed using RNA STAR [25]. The mapping percentage ranged from 93.2% to 94.3%, and the number of uniquely mapped reads varied between 11.1 and 12.7 million, depending on the sample.

In order to measure gene expression, featureCounts [26] was used to obtain the counts reads mapped per gene using the annotated reference genome (also obtained from the Pubmed genome database). The assignment of reads per sample ranged from 20.9 to 23.8 million, with a percentage of assignments varying between 86.5% and 88.8%.

The normalization of the data and the differential gene expression analysis were carried out using edgeR [27].

#### Gene enrichment analysis

goseq [28] was used to carry out a gene enrichment analysis of the RNA counts. This particular tool considers length bias in order to perform the gene enrichment and was used to identify significantly differentially expressed genes within both using Gene Ontology (GO) terms and Kyoto Encyclopedia of Genes and Genomes (KEGG) pathways. To determine statistical significance, filters requiring each group (GO:Term or KEGG pathway) to consist of a minimum of four genes and have a false discovery rate (FDR) < 0.05 were applied. Detailed information regarding each identifier specific to *Y. lipolytica* was obtained from the Gene Ontology and KEGG websites (http://geneontology.org/ and KEGG: Kyoto Encyclopedia of Genes and Genomes).

## Results and discussion

### ALE of *Y. lipolytica* ACA DC 50109 for an efficient hexanoic acid assimilation

Hexanoic acid is often obtained as a result of anaerobic fermentation of certain organic wastes [15]. Given that this acid is the most inhibitory of all SCFAs for *Y. lipolytica* [29], it is of utmost importance to obtain *Y. lipolytica* strains able to consume high concentrations of hexanoic acid.

In recent years, the routes of assimilation of sugars (*de novo* assimilation) have been widely studied in yeasts [6]. However, the assimilation of a variety of SCFAs, such as hexanoic acid, remains unknown. Therefore, it is not possible to follow a targeted engineering strategy to improve hexanoic acid consumption. In this regard, ALE has been established as a good option to achieve this, as it allows an unidentified genetic trait to be self-optimized [19]. By applying this methodology, strains with a desired phenotype, e.g., increased tolerance against multiple stressors, faster growth rates, etc. can be obtained [20].

**Fig. 1:**
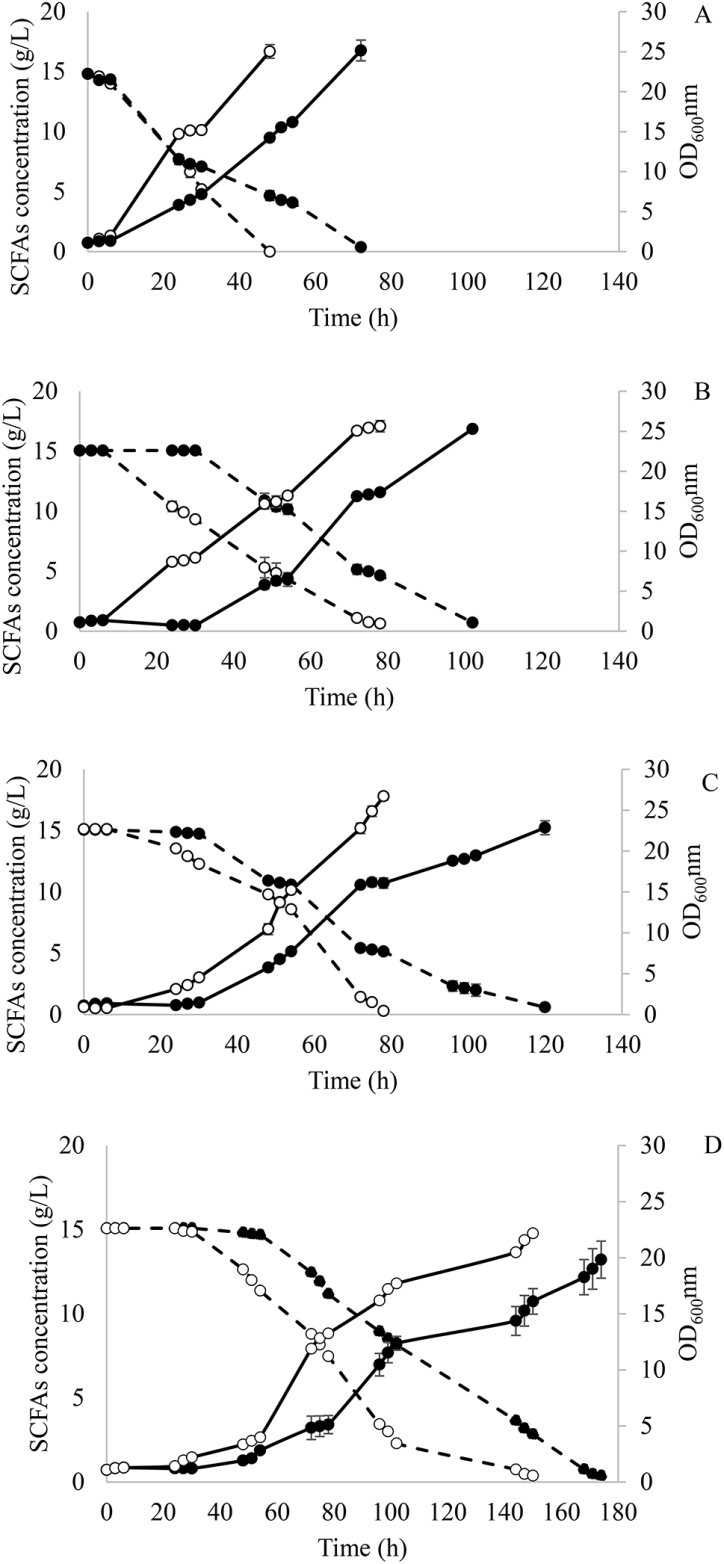
Fermentation course of *Y. lipolytica* in the first (●) and last adaptation round (○) at **(A)** acetic:hexanoic ratio 1:1 (SM 1), **(B)** 1:2 (SM 2), **(C)** 1:4 (SMa 3) and **(D)** 1:6 (SM 4) during adaptive laboratory evolution. Biomass growth and SCFAs consumption are represented as — and --, respectively

Evolved cells from the first and last adaptation round in each media with different A:H were compared. Time courses for cell growth and SCFAs consumption are shown in Fig. 1. *Y. lipolytica* in SM 1 showed almost no lag phase. However, there was a 1.5-fold increase in SCFAs consumption rate and 1.8-fold higher growth rate when comparing cells obtained after 1 round and 9 rounds of adaptation in SM 1 (Fig. 1A). Nevertheless, the evolved strain suffered inhibition when it was transferred to SM 2, as evidenced by the 30-h lag phase (Fig. 1B). Once the cells got adapted to higher hexanoic acid concentrations during continuous rounds of adaptation, the lag phase was significantly reduced to 6 h. When comparing cells obtained in rounds 10 and 18 in SM 2, the substrate consumption and growth rate were 2.0 and 2.8-fold higher, respectively (Fig. 1B).

Similar results were obtained when cells were transferred to SM 3. A reduction in the lag phase from 30 h to 6 h was observed between adaptation rounds 19 and 27. Moreover, *Y. lipolytica* achieved a SCFAs consumption and growth rate 1.1- and 1.8-fold higher during the first 48 h of fermentation when cells from rounds 19 and 27 were compared (Fig. 1C).

To confirm that there was still some room for improving the tolerance to higher hexanoic acid concentrations, *Y. lipolytica* was further adapted to a medium with an A:H ratio of 1:6 (SM 4). After 9 additional adaptation rounds, the strain was able to reduce its lag phase from 30 h to 24 h and showed consumption and growth rates 1.2- and 1.8-fold higher when comparing cells from rounds 28 and 36 (Fig. 1D).

**Fig. 2:**
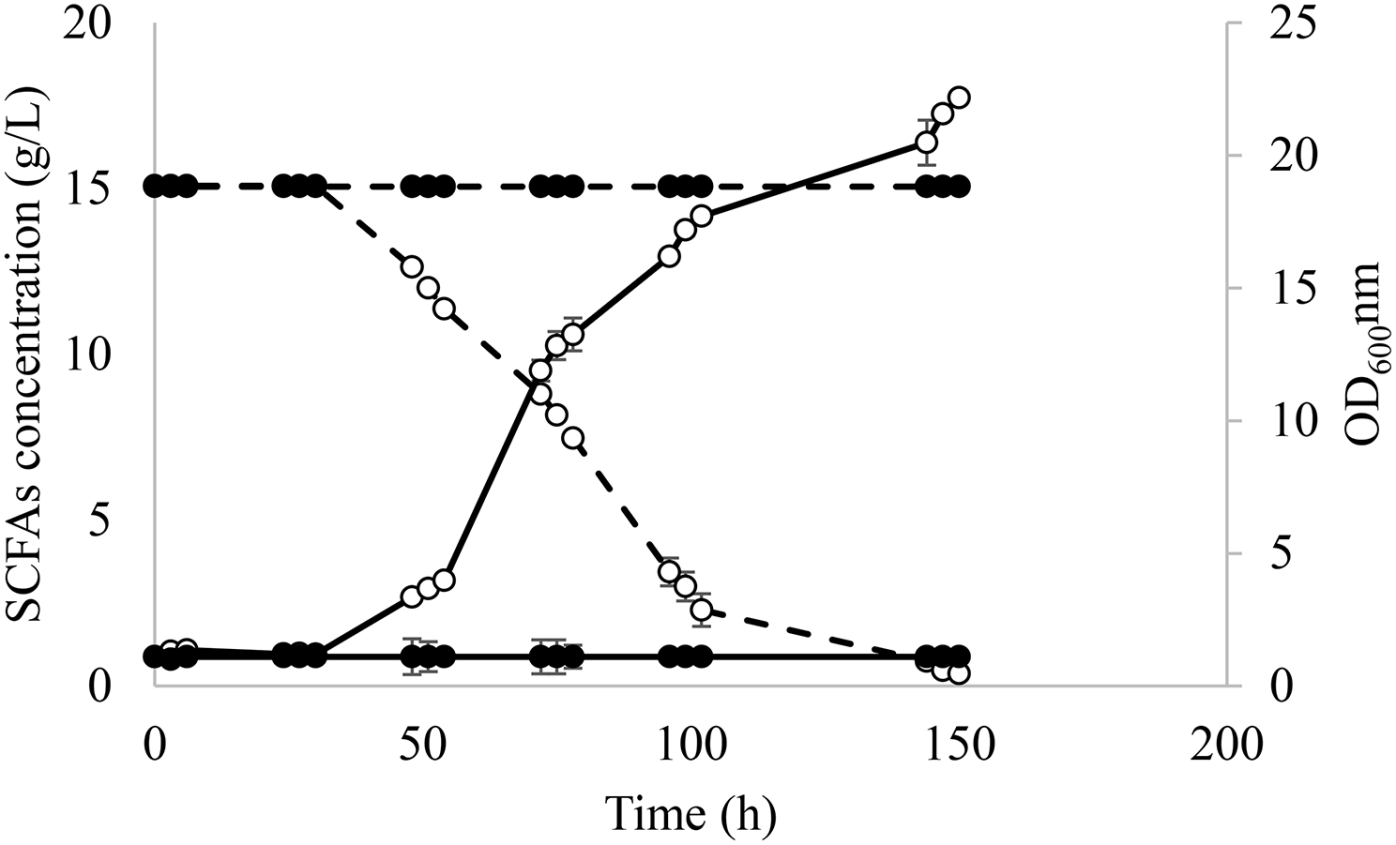
Fermentation course for evolved *Y. lipolytica* (○) and wild type strains (●) in SM 4 containing acetic:hexanoic ratio of 1:6. Biomass growth and SCFAs consumption are represented as — and --, respectively

The evolved and WT strains were tested in media with A:H 1:6. As it is shown in Fig. 2, only the evolved strain was able to grow on this medium containing the highest concentration of hexanoic acid (6.24 g/L), which corroborated that the strain adaptation was exclusively due to changes in the genotype since phenotypic adaptations are known to be lost after growing the strains on rich media as YPD [30]. Due to the absence of growth of the WT in SM 4 and for comparative purposes, the clone selection and fermentations for transcriptomic analysis were performed in SM 3.

**Fig. 3:**
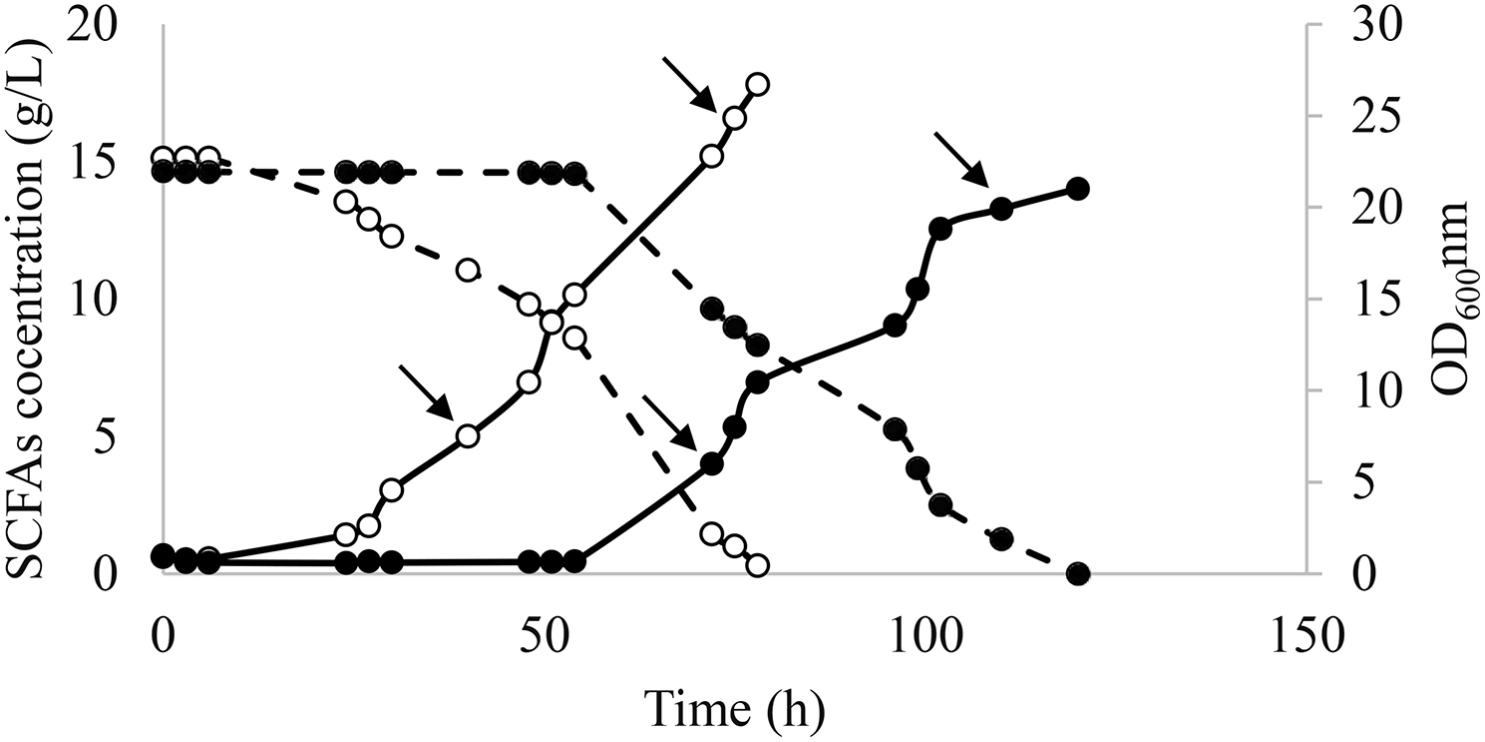
Fermentation course for evolved *Y. lipolytica* (○) and wild type strains (●) in SM 3 containing acetic:hexanoic ratio of 1:4. Biomass growth and SCFAs consumption are represented as — and --, respectively. The arrows represent the timepoints when the samples for RNAseq were taken

After the selection of the best colony as described in Sect. 2.4, a preinocula of the evolved and WT strains were prepared in YPD and used in fermentation in SM 3 (Fig. 3). As expected, the evolved strain exhibited a shorter lag phase than the WT strain (6 h instead of 54 h). After 72 h of fermentation, the evolved strain showed 2.5- and 3.4-fold higher SCFAs consumption and growth rate, respectively, when compared to the WT (Fig. 3). These results demonstrated the genotypic changes leading to improvements in both; cells growth and carbon source consumption. Samples for RNA sequencing were taken at 40 h and 75 h for the evolved strain and at 70 h and 110 h for the parental strain. These time points corresponded to the beginning and the end of the exponential phase.

### Transcriptomic changes of *Y. lipolytica* in response to high concentrations of hexanoic acid

**Fig. 4:**
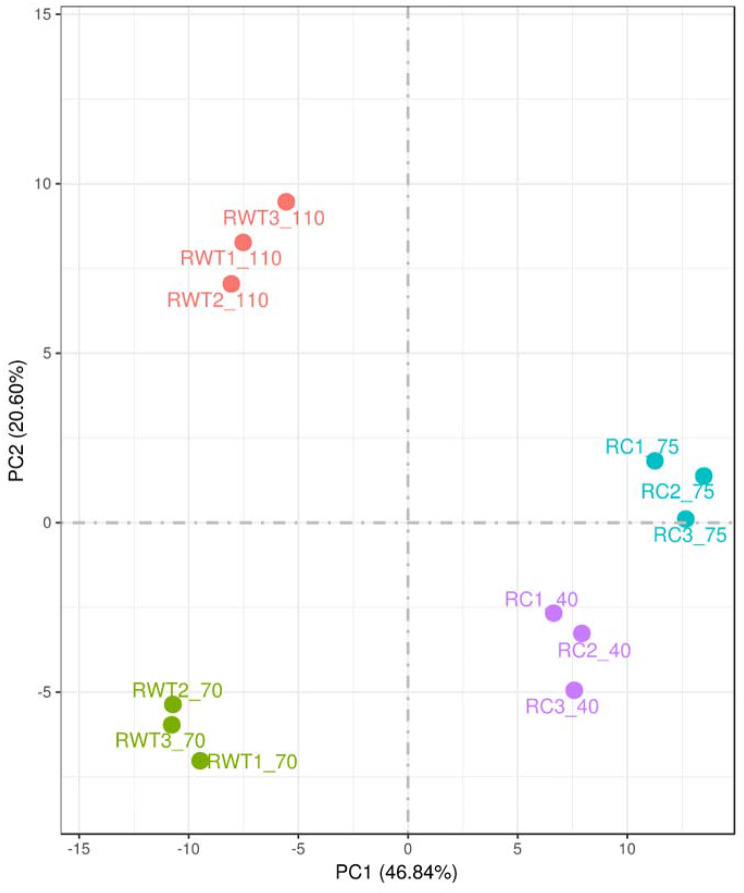
Principal component analysis (PCA) of the wild type and evolved strain at the beginning (green and purple, respectively) and at the end (red and blue, respectively) of the exponential phase

**Fig. 5:**
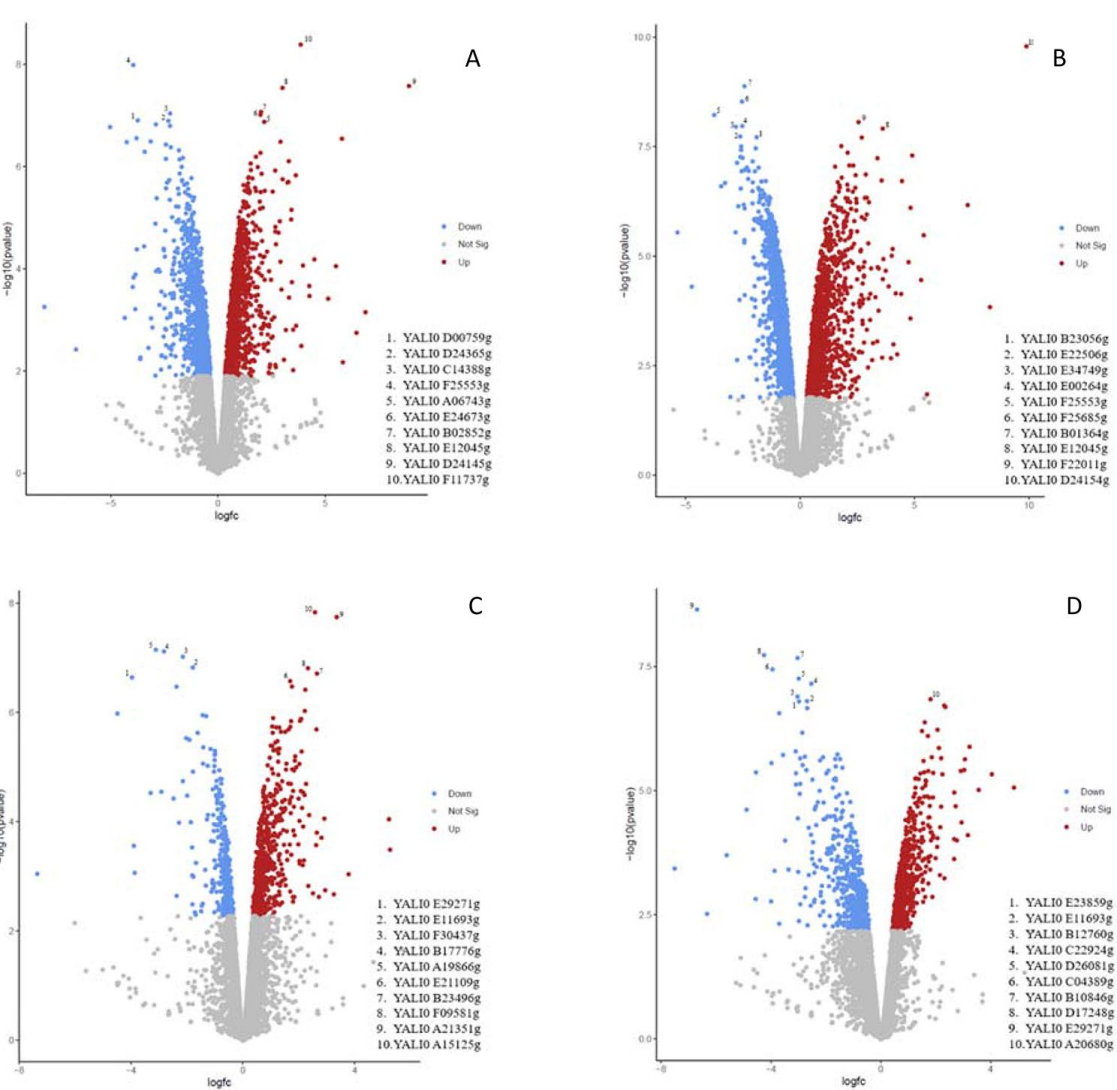
Volcano plots representing up- (red) and down-regulated (blue) genes in Evo-B vs. WT-B **(A)**, Evo-E vs. WT-E **(B)**, Evo-B vs. Evo-E **(C)** and WT-B vs. WT-E **(D)**. The 10 most significant genes are tagged in each figure

The Principal Component Analysis (PCA) based on the gene expression profile of the samples showed that the evolved strain was grouped in two sets of clusters clearly differentiated by the time of fermentation at which the sample was taken. Likewise, the WT formed two separate clusters for each sample-time and were different to the ones from the evolved strain (Fig. 4). This indicated that, in terms of the gene expression profile, differences were observed both between the evolved and WT strains, as well as between the different fermentation time points. To deepen investigate the differences between the individual gene expression profiles, four comparisons were performed: (i) evolved vs. WT strains at the beginning of the exponential phase (40 h vs. 70 h) (Evo-B vs. WT-B) (ii) evolved vs. WT strains at the end of the exponential phase (75 h vs. 110 h) (Evo-E vs. WT-E), (iii) the beginning and the end of the exponential phase of the evolved strain (40 h vs. 75 h) (Evo-B vs. Evo-E) and, (iv) the beginning and the end of the exponential phase of the WT strain (70 h vs. 110 h) (WT-B vs. WT-E). Differential gene expression analysis (FDR < 0.05) revealed that out of 6404 genes investigated, 1111 and 980 were significantly up- and down-regulated, respectively in Evo-B vs. WT-B, 1375 and 1420 were up- and down-regulated in Evo-E vs. WT-E, 514 and 376 were up- and down-regulated in Evo-B vs. Evo-E and 602, and 510 were up- and down-regulated in WT-B vs. WT-E (Fig. 5).

In order to identify those differentially expressed genes, two databases were used: UniProt (https://www.uniprot.org/) and NCBI (https://www.ncbi.nlm.nih.gov/). Nevertheless, disparities were found between the gene annotations recorded in each database for *Y. lipolytica* CLIB122 strain. Finally, genes were identified using NCBI, since it was the database that presented the most information on this yeast. It should be noted that even so, 43.8% of all the genes included in the analysis belonged to the category of unidentified genes.

Specifically, only those genes that showed up- and down-regulation between − 2 < logFC > 2 in each comparison were considered. In addition, RNA-seq enrichment analyses of each comparison referred to GO:Terms and KEGG pathways were carried out, which allowed to determine more precisely where the key points of regulation occurred in each case. Finally, in order to deepen the analysis and in view of the fact that genes related to membrane, stress and lipids were the most highly regulated, all genes related to these terms that showed high expression (logCPM > 10) were analyzed.

#### Importance of robustness and survival gene expression at the beginning of the exponential phase

During the analysis of Evo-B vs. WT-B, 162 genes that exhibited differential up- and down-regulation within a -2 < logFC > 2 were identified. Furthermore, 13 GO:Terms and 10 KEGG pathways (padj over-represented < 0.05) that contained a moderate-to-high number of up- and down-regulated genes were observed. Finally, 3 stress- and lipid-related genes that were up-regulated with a logCPM > 10 were found (Supplementary Table 1).

As shown in Fig. 3, the evolved strain reached an OD 1.7-fold higher than the WT. Moreover, genes involved in the mitochondrial respiratory chain, the mitochondrial electron transport as well as the TCA cycle activity were found to be up-regulated. These functions are known to be related to enhanced cellular respiration and growth [31], which would explain the improved growth of the evolved strain at the beginning of the exponential phase. The evolved strain also presented the up-regulation of YALI0 E19448g gene (logFC 2.6), which is expressed under oxidative stress conditions. Furthermore, the high expression of the up-regulated YALI0 C03443g gene (logCPM 12.0 and logFC 0.6), which is activated in response to hydrogen peroxide, in order to eliminate toxic forms of oxygen as a result of the increased cellular respiration, was observed [32]. These results indicated that the evolved strain activated a greater number of genes to improve its growth in SM 3, compared to the WT.

Remarkably, up-regulation of carbon catabolite repression (CCR) was also observed in the evolved strain (Supplementary Table 1). This is a transcriptional regulatory phenomenon of bacteria, fungi and yeast that coordinate the expression of genes required for preferential utilization of carbon sources [33]. Since carbon metabolism is essential for growth, CCR regulation is critical for the robustness of microorganisms. It can be thus highlighted that the evolved strain presented a more robust mechanism for the assimilation of SCFAs compared to the WT. The evolved strain presented a high expression over the up-regulated YALI0 D17864g (logCPM 10.5 and logFC 0.6), which codes for FAA1 and long-chain fatty acid-CoA ligase activity [34]. Since the long-chain fatty acid-CoA ligase activity is used to consume carbon sources from C6 to C20 [35], it could be stated that the evolved strain increased the expression of this gene to efficiently metabolize the hexanoic acid in SM 3. Moreover, the down-regulation of glyoxylate and dicarboxylate metabolism, which is used by microorganisms to assimilate C2 carbon compounds (such as acetic acid), was observed. This indicates that the evolved strain did not need to activate this route as much as the WT to grow since it activates more specific routes to assimilate the rest of the acids. When the KEGG pathways analysis was performed (Supplementary Table 1), up-regulated genes related to oxidative phosphorylation were found (KEGG:yli00190). These results, together with the aforementioned, confirmed that the evolved strain exhibited a mechanism to enhance growth, increase cellular respiration and divert carbon source to energy when compared to the WT.

Diffusion of SCFAs into the yeast can lead to membrane damage [36]. The higher growth inhibition caused by longer chain SCFAs could be also linked to greater membrane damage during their uptake [29]. Genes responsible of membrane integrity (ARP2/3 protein complexes and ergosterol biosynthesis) [37, 38] were found to be upregulated in the evolved strain, resulting in reduced membrane damage caused by SCFAs. Furthermore, the high expression of the up-regulated YALI0 C11341g gene (logCPM 11.7 and logFC 0.7), which operates at the plasma membrane level, indicated that lipid metabolism was activated towards the formation of membrane lipids. In addition, the evolved strain presented up-regulation of genes related to fatty acid biosynthesis, indicating that in early stages this strain was prepared to synthesize membrane lipids or accumulate them as triglycerides. It is worth mentioning that the evolved strain also exhibited up-regulation of steroid synthesis. The synthesis of these compounds in yeast helps the microorganism to improve its ability to survive in unfavorable or inhibitory growth conditions [39]. Therefore, steroid synthesis was activated by yeast cells to improve its survival in the presence of a high concentration of hexanoic acid.

Iron and zinc transport genes were found to be down-regulated in the evolved strain compared to the WT (Supplementary Table 1). Likewise, small unit processome, biosynthesis of amino acids and their transport were down-regulated. An increased growth of the evolved strain should result in increased activity of these genes, as they are related to cell division and growth [40]. Nevertheless, the evolved strain optimized its growth in SM 3 to such an extent that it did not need to activate these genes to the levels at which they were activated in the WT (in an unfavorable environment).

#### Major changes over gene expression of butyric and hexanoic acids assimilation, starvation genes and lipid production at the end of the exponential phase

The analysis of Evo-E vs. WT-E revealed a total of 182 genes differentially up- and down-regulated with a -2 < logFC > 2. Additionally, 27 GO:Terms and 16 KEGG pathways with a moderate-to-high number of up- and down-regulated genes were identified (padj overrepresented < 0.05). Lastly, 8 transport and lipids-related genes that presented a differential expression with a logCPM > 10 were observed (Supplementary Table 2).

At the end of the exponential phase, the enhanced growth of the evolved strain over the WT was observed in the up-regulation of genes with mitotic functions, microtubule binding, microfilament motor activity and cytokinesis, all of them related to cell division [41]. There was also an up-regulation of genes in charge of iron transport, which also implied an improvement in growth [42]. Moreover, KEGG pathways analysis revealed the up-regulation of the cell cycle (KEGG:yli04111) and steroid biosynthesis (KEGG:yli00100) which, as expected, showed that the evolved strain presented better growth and improved cell tolerance than the WT during the whole fermentation. Peroxisome function (KEGG:yli04146), which is involved in the β-oxidation of fatty acids [43], was found to be up-regulated in the evolved strain. Likewise, the up-regulation of enoyl-CoA hydratase activity gene (YALI0 F28567g) (logFC 2.3), which is related to the degradation of compounds ranging from C4 to C16 (a range that includes butyric, valeric and hexanoic acid) [44, 45], was also observed. These results, together with the down-regulation of the glyoxylate and dicarboxylate pathway, and with the expression of the YALI0 D17864g gene observed in Evo-B vs. WT-B, confirmed that the evolved strain had adjusted its cell mechanisms to better assimilate longer SCFAs than acetic acid.

As observed during the beginning of the exponential phase, genes responsible for enhancing membrane integrity (ergosterol biosynthetic processes, actin filament binding, N-glycan biosynthesis, terpenoid biosynthesis and inositol phosphate biosynthesis) [38] were also found up-regulated in the evolved strain at the end of the exponential phase. This indicates that the evolved strain was able to avoid cell damage caused by the diffusion of SCFAs, and maintained a robust cell membrane throughout the fermentation, unlike the WT.

Interestingly, the evolved strain showed the up-regulation of the YALI0 B21582g gene (logFC 2.1), which carries out the response to cell starvation. Since the evolved strain at the end of the exponential phase had less substrate left in the medium than the WT (Fig. 3), the up-regulation of this particular gene may serve as a signal to identify that the carbon source was limited. Thus, the yeast would have to enter the stationary phase. In this sense, down-regulation of the gene responsible for the response to hydrogen peroxide (YALI0 E34749g) (logFC − 2.8), as well as the carbohydrate transport gene (YALI0 F25553g) (logFC − 3.7), both related with yeast cell growth, were also found significantly expressed in the evolved strain. As a result of not being able to cell duplication, down-regulation of ribosome and RNA polymerase, related to RNA and protein synthesis from DNA sequences [46], was also observed. These results corroborated the aforementioned relation between a low carbon source availability and decreased cell growth, resulting in the yeast cell adaptation to enter the stationary phase.

**Fig. 6:**
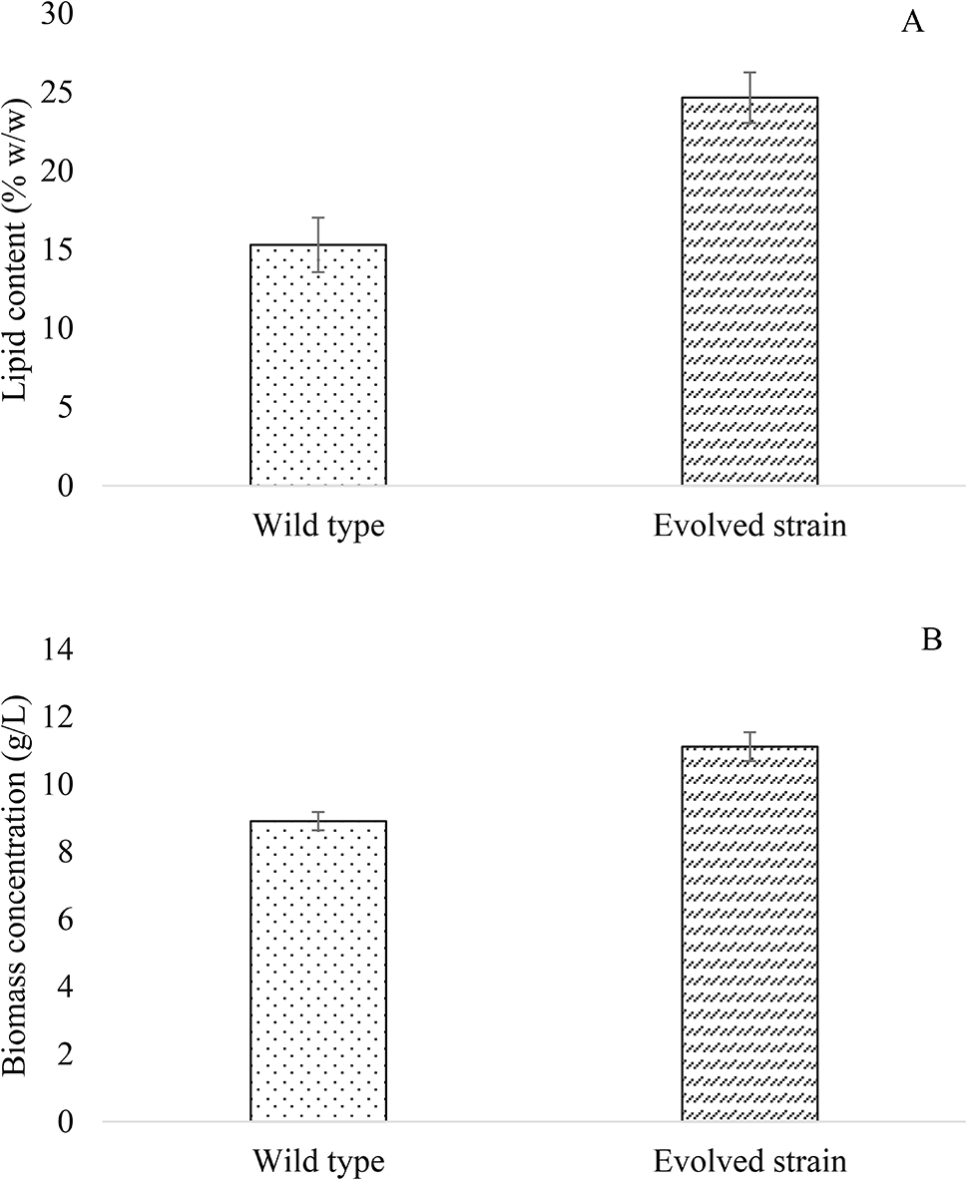
Lipid content **(A)** and biomass concentration **(B)** attained by *Y. lipolytica* evolved and wild type strains in SM 3 containing acetic:hexanoic ratio of 1:4

It should be noted that the evolved strain presented a high expression of the up-regulated gene YALI0 E16016g (logCPM 10.5 and (logFC 1.6), which codes for FAT1 and is related to lipid droplets formation and is responsible for mobilizing long-chain acids within the cell [47]. This would indicate that the evolved strain could accumulate more lipids than the WT when growing in the same culture medium. To confirm this, lipid quantification from both, the evolved and WT strains, was carried out at the end of the exponential phase. As shown in Fig. 6, there was a 1.6-fold increase in lipid content in the evolved strain compared to the WT, which corroborated that the observed up-regulation of YALI0 E16016g implied an increase in lipid accumulation. Although the adaptation of *Y. lipolytica* was aimed to improve its tolerance and growth in media containing high concentrations of hexanoic acid, the yeast also managed to optimize lipid production from this carbon source. This fact corroborates that ALE is an efficient strategy to obtain beneficial secondary improvements without the need to know the exact pathways involved.

#### Energy management and α-linoleic acid production as key gene expression for growth of the evolved strain

Throughout Evo-B vs. Evo-E analysis, 51 genes showing differential up- and down-regulation with a -2 < logFC > 2 were found. Furthermore, 11 GO:Terms and 18 KEGG pathways (padj over represented < 0.05) with moderate-to-high number of up- and down-regulated genes were identified. Finally, 6 transport and lipid-related genes that were up- and down-regulated with a logCPM > 10 were detected (Supplementary Table 3).

Several genes involved in cell division (cell cycle, cellular bud neck, microtubule motor activity, kinesin complex, microtubule binding, microtubule-based movement, mitotic chromosome condensation and microtubule cytoskeleton organization) [41] were found to be up-regulated in the evolved strain at the end of the exponential phase. This can be related to an increase in cellular respiration, through the up-regulation of peroxisome activity, β-oxidation of fatty acids and oxidoreductase activity, and biosynthesis of amino acids. As already known, all these genes are related to cell growth [31, 43], so their up-regulation lead to an increase in cell growth as shown in Fig. 3. Moreover, up-regulation of steroid biosynthesis was observed, indicating that the evolved strain increased the amount of this compound throughout the fermentation to maintain the viability and fermentation activity of the cells. Interestingly, yeast cells also over-expressed genes involved in α-linoleic acid synthesis. Yazawa et al. (2009) [48] observed that *Saccharomyces cerevisiae*, which produces linoleic and α -linolenic acids, showed an alkaline pH-tolerant phenotype. It is worth mentioning that the pH was not adjusted during fermentation in this study (Sect. 2.3). In fact, pH 9 was reached at the end of the exponential phase as a result of the consumption of SCFAs in SM 3. In this sense, the evolved strain could modify its cell mechanisms to develop a phenotype that favored its growth in alkaline media.

At the end of the exponential phase, with a low carbon source, it can be assumed that the evolved strain would have a limited ability to grow. Therefore, the yeast cells repressed genes related to active transport of nutrients. In addition, the repression of the plasma membrane lipid binding gene (YALI0 D20526g) and GO:0005886 (plasma membrane) also indicates that the evolved strain drove the energy from growth and membrane lipids synthesis for survival. These results, together with those observed at Evo-E vs. WT-E, confirmed that the evolved strain could be adapting its cell mechanisms to enter already in the stationary phase. Remarkably, the down-regulation of the acetyl-CoA biosynthetic process from pyruvate was also observed (Supplementary Table 3). Since pyruvate is a compound derived from glucose degradation, the repression of the GO:0006086 by the evolved strain indicates that the yeast cells identified the available carbon source in SM 3 was different from sugars, and therefore repressed cell mechanisms that will no longer used to save energy.

#### Gene expression of WT strain throughout the fermentation confirms the greater ease to assimilate acetic acid over other SCFAs

After the analysis of WT-B vs. WT-E, a total of 101 genes that exhibited differential up- and down-regulation with a range of -2 < logFC > 2 were found. In addition, 8 GO:Terms and 15 KEGG pathways with a moderate-to-high number of up- and down-regulated genes (padj overrepresented < 0.05) were observed. Finally, 2 down-regulated transport-related genes with a logCPM > 10 were identified (Supplementary Table 4).

Throughout the fermentation of the WT strain, the up-regulation of the mitochondrial respiratory chain function (GO:0005747) and oxidative phosphorylation processes (KEGG:yli00190) was observed. In addition, the up-regulation of RNA replication, biosynthesis of amino acids and their transport, which as previously mentioned are directly related to yeast growth and division, was also identified. Nevertheless, it should be noted that only 1 g/L of SCFAs was available at the end of the exponential phase (Fig. 3). Due to this limitation that prevented the yeast from continuing to grow, the transport of different nutrients (zinc, copper and iron), nucleotide metabolism, synthesis of some non-necessary amino acids (purine and pyrimidine), DNA replication and homologous recombination were repressed. This would be expected due to the scarcity of carbon source and nutrients in SM 3 at the end of the exponential phase. Nevertheless, it is noteworthy that in the case of the evolved strain, only down-regulation of nutrient transport genes and the TCA cycle occurred. This indicates that the evolved strain was able to manage resources more efficiently and repressed only the necessary genes to adapt to the conditions found in media with A:H 1:4 (SM 3). Furthermore, the down-regulation of genes related to alternative oxidase activity was observed in the WT strain, indicating that it also grows worse than the evolved strain based on the results observed above.

Interestingly, mainly acetic acid transport genes as well as other carbohydrate transporters with no specific function were over-expressed in the WT strain. The fact that the WT strain activated a higher number of acetic acid transporter genes than those observed in the evolved strain, confirmed that this acid (the one with the shortest carbon chain) was the most easily assimilated by *Y. lipolytica* prior to any adaptation. The other up-regulated carbohydrate transporters could be used by the yeast throughout the fermentation to assimilate the rest of the acids in the most efficient way. The repression of the glycerophospholipids synthesis, which helps to improve cell membrane robustness [49], was also observed. This indicated that the WT strain did not allocate resources to keep its membrane undamaged by SCFAs diffusion, contrary to what was observed in the evolved strain.

## Conclusions

ALE enabled the obtaining of an evolved strain of *Y. lipolytica* capable of assimilating high concentrations of hexanoic acid in a mixture of SCFAs. The adaptation towards high concentrations of hexanoic acid increased yeast robustness and led to the optimization of SCFAs assimilation. The transcriptomic analysis revealed the up-regulation of CCR which increased yeast robustness. Remarkably, there was an improvement in lipid production over the WT strain. These results provide new insights related to the use of cheap effluents with high concentrations of SCFAs as an interesting alternative to improve the economic viability of the lipid production process.

### Electronic supplementary material

Below is the link to the electronic supplementary material.

Supplementary Material 1

## Data Availability

RNAseq used in this study can be found in NCBI (https://www.ncbi.nlm.nih.gov/) under the accession number PRJNA1035025(https://www.ncbi.nlm.nih.gov/sra/PRJNA1035025).
