# Supplementary material for: Transcriptomic profiling of an evolved Yarrowia lipolytica strain: tackling hexanoic acid fermentation to increase lipid production from short-chain fatty acids
Source: Microb Cell Fact. 2024 Apr 3;23:101. doi: 10.1186/s12934-024-02367-4 (PMC10988856; doi:10.1186/s12934-024-02367-4)
Supplement: Supplementary file 1 — Supplementary Material 1 [file 12934_2024_2367_MOESM1_ESM.docx]

**Supplementary Table 1.** Significant up- or down-regulated genes within a -2<logFC>2 obtained from the differential analysis. GO:Terms, KEGG pathways (padj<0.05) and high expression genes (logCPM>10) identified through Evo-B vs. WT-B are included in this table. Up-regulation is represented in red while down-regulation in blue. Information about the corresponding gene product was obtained from NCBI database at ([NCBI](https://www.ncbi.nlm.nih.gov/)).

**ID Description/Function logFC padj logCPM**

| YALI0 F06666g | Unknown function | 2.00 | - | - |
| --- | --- | --- | --- | --- |
| YALI0 B00528g | Transcription factor TFIIIC complex | 2.01 | - | - |
| YALI0 F26015g | Unknown function | 2.02 | - | - |
| YALI0 C02915g | THO complex part of transcription export complex | 2.03 | - | - |
| YALI0 E07139g | Nuclear export signal receptor activity | 2.03 | - | - |
| YALI0 B17270g | Anaphase-promoting complex binding | 2.04 | - | - |
| YALI0 F27533g | Component of the INO80 complex which remodels chromatin by shifting nucleosomes and is involved in DNA repair (By similarity) | 2.04 | - | - |
| YALI0 E05709g | Unknown function | 2.05 | - | - |
| YALI0 A00847g | Putative potassium channel subunit | 2.06 | - | - |
| YALI0 D17996g | DNA binding. bending | 2.06 | - | - |
| YALI0 A20086g | Demethylates the phosphatase PP2A catalytic subunit | 2.07 | - | - |
| YALI0 C11946g | Exocyst complex protein EXO70 | 2.09 | - | - |
| YALI0 C04620g | mRNA binding | 2.10 | - | - |
| YALI0 C01331g | Regulation of translational initiation | 2.12 | - | - |
| YALI0 F15741g | Unknown function | 2.13 | - | - |
| YALI0 A14839g | Involved in intrinsic apoptotic signaling pathway in response to endoplasmic reticulum stress | 2.14 | - | - |
| YALI0 B21230g | hexose transmembrane transporter activity | 2.14 | - | - |
| YALI0 F07194g | Unknown function | 2.16 | - | - |
| YALI0 C15092g | Unknown function | 2.17 | - | - |
| YALI0 E28578g | Unknown function | 2.18 | - | - |
| YALI0 B03190g | Sphingolipid biosynthetic process | 2.18 | - | - |
| YALI0 F09669g | Translational elongation | 2.20 | - | - |
| YALI0 E34001g | Protein phosphorylation | 2.22 | - | - |
| YALI0 D22957g | Proteolysis | 2.29 | - | - |
| YALI0 C24398g | DNA binding. bending | 2.29 | - | - |
| YALI0 F28567g | Enoyl-CoA hydratase activity | 2.32 | - | - |
| YALI0 B00858g | DNA binding. bending | 2.33 | - | - |
| YALI0 F27313g | Mitochondrial inner membrane lipid binding | 2.33 | - | - |
| YALI0 F15037g | Unknown function | 2.34 | - | - |
| YALI0 C08613g | Unknown function | 2.37 | - | - |
| YALI0 E30481g | Glutamate-5-semialdehyde dehydrogenase activity | 2.38 | - | - |
| YALI0 D24321g | Palmitoyl-(protein) hydrolase activity | 2.38 | - | - |
| YALI0 E13343g | 2-oxoglutarate-dependent dioxygenase activity | 2.39 | - | - |
| YALI0 F30349g | Sulfate adenylyltransferase (ADP) activity\|bis(5'-nucleosyl)-tetraphosphatase activity | 2.39 | - | - |
| YALI0 C11319g | Unknown function | 2.41 | - | - |
| YALI0 E09955g | Sin3-type complex | 2.44 | - | - |
| YALI0 A13673g | Unknown function | 2.48 | - | - |
| YALI0 B07381g | Mitotic sister chromatid segregation | 2.50 | - | - |
| YALI0 B20108g | Cytoplasm | 2.52 | - | - |
| YALI0 B20570g | Unknown function | 2.54 | - | - |
| YALI0 B21714g | Unknown function | 2.55 | - | - |
| YALI0 B13156g | Protein folding | 2.55 | - | - |
| YALI0 E19448g | Cellular response to oxidative stress | 2.62 | - | - |
| YALI0 F23199g | Unknown function | 2.67 | - | - |
| YALI0 A14234g | CoA-ligase activity | 2.68 | - | - |
| YALI0 E32065g | Ergosterol biosynthetic process | 2.68 | - | - |
| YALI0 C08701g | Involved in non-homologous end joining (NHEJ) DNA double strand break repair | 2.68 | - | - |
| YALI0 E00726g | Mitotic cell cycle | 2.70 | - | - |
| YALI0 C00649g | Unknown function | 2.71 | - | - |
| YALI0 A18656g | Dolichol-phosphate-mannose synthase complex | 2.83 | - | - |
| YALI0 D25014g | Unknown function | 2.89 | - | - |
| YALI0 E16907g | Choline kinase activity | 2.92 | - | - |
| YALI0 D27060g | Unknown function | 2.92 | - | - |
| YALI0 F24475g | V-type proton ATPase proteolipid subunit | 2.97 | - | - |
| YALI0 A03751g | mRNA binding | 3.01 | - | - |
| YALI0 E21890g | DNA 5'-adenosine monophosphate hydrolase activity | 3.02 | - | - |
| YALI0 D10549g | Dolichyl-phosphate-mannose-protein mannosyltransferase activity | 3.08 | - | - |
| YALI0 F18304g | Unknown function | 3.17 | - | - |
| YALI0 F23221g | Threonine synthase activity | 3.24 | - | - |
| YALI0 C21604g | Serine-type carboxypeptidase activity | 3.29 | - | - |
| YALI0 E16555g | TRAPPIII protein complex | 3.31 | - | - |
| YALI0 A18381g | Rho GDP-dissociation inhibitor activity | 3.32 | - | - |
| YALI0 B01716g | Peptide-alanine-α-N-acetyltransferase activity | 3.42 | - | - |
| YALI0 B02728g | (2R)-2-phosphoglycerate = (2R)-3-phosphoglycerate | 3.43 | - | - |
| YALI0 B19844g | Unknown function | 3.45 | - | - |
| YALI0 E34170g | Arp2/3 protein complex | 3.51 | - | - |
| YALI0 F01100g | Unknown function | 3.51 | - | - |
| YALI0 A20570g | Fluoride transmembrane transport | 3.63 | - | - |
| YALI0 D25102g | COP9 signalosome | 3.65 | - | - |
| YALI0 E25179g | Transmembrane transporter activity | 3.86 | - | - |
| YALI0 F30107g | Unknown function | 3.89 | - | - |
| YALI0 D05995g | Phosphatidylinositol dephosphorylation | 3.97 | - | - |
| YALI0 C10780g | Mitochondrial translation | 4.26 | - | - |
| YALI0 E02002g | Mitochondrion | 4.27 | - | - |
| YALI0 E32879g | Carbon catabolite repression of transcription | 4.50 | - | - |
| YALI0 D23727g | Unknown function | 5.14 | - | - |
| YALI0 A13233g | Unknown function | 5.50 | - | - |
| YALI0 B09053g | mRNA splicing. via spliceosome | 5.78 | - | - |
| YALI0 D06589g | Protein N-linked glycosylation via asparagine | 5.83 | - | - |
| YALI0 F30767g | Involved in inositol deacylation of GPI-anchored proteins | 6.46 | - | - |
| YALI0 D25652g | Spliceosomal complex assembly | 6.88 | - | - |
| YALI0 A07128g | Unknown function | 8.91 | - | - |
| YALI0 A15906g | Alditol:NADP+ 1-oxidoreductase activity\|cytosol | -2.01 | - | - |
| YALI0 B09625g | Proline catabolic process to glutamate | -2.02 | - | - |
| YALI0 E28622g | Urea transmembrane transporter activity | -2.03 | - | - |
| YALI0 E22506g | Amino acid transmembrane transport | -2.05 | - | - |
| YALI0 E33363g | Aspartic-type endopeptidase activity | -2.06 | - | - |
| YALI0 B00660g | Regulation of transcription by RNA polymerase II | -2.06 | - | - |
| YALI0 B21868g | Unknown function | -2.06 | - | - |
| YALI0 C07821g | Unknown function | -2.06 | - | - |
| YALI0 E27984g | Unknown function | -2.07 | - | - |
| YALI0 D08382g | Glycerophosphodiester transmembrane transport | -2.07 | - | - |
| YALI0 B23474g | Unknown function | -2.07 | - | - |
| YALI0 A10967g | Unknown function | -2.12 | - | - |
| YALI0 E34881g | Phosphatidic acid biosynthetic process | -2.12 | - | - |
| YALI0 E31515g | Triglyceride lipase activity | -2.14 | - | - |
| YALI0 C23661g | Serine-type carboxypeptidase activity | -2.14 | - | - |
| YALI0 C21252g | Unknown function | -2.15 | - | - |
| YALI0 F01386g | Iron ion transport | -2.17 | - | - |
| YALI0 C18095g | Unknown function | -2.19 | - | - |
| YALI0 D08052g | Serine-type carboxypeptidase activity | -2.19 | - | - |
| YALI0 B01364g | Unknown function | -2.20 | - | - |
| YALI0 B03344g | Sulfate transport | -2.21 | - | - |
| YALI0 A19866g | Unknown function | -2.21 | - | - |
| YALI0 C07634g | Unknown function | -2.21 | - | - |
| YALI0 B19976g | Formate dehydrogenase (NAD+) activit | -2.22 | - | - |
| YALI0 C14388g | Methyltransferase activity | -2.23 | - | - |
| YALI0 B10846g | Iron ion transport | -2.23 | - | - |
| YALI0 F13607g | Transmembrane transporter activity | -2.24 | - | - |
| YALI0 F01320g | Unknown function | -2.26 | - | - |
| YALI0 A02783g | Unknown function | -2.26 | - | - |
| YALI0 F19712g | Mitochondrial ATP transmembrane transport | -2.27 | - | - |
| YALI0 D24365g | Unknown function | -2.31 | - | - |
| YALI0 E10197g | Unknown function | -2.36 | - | - |
| YALI0 F13849g | Unknown function | -2.37 | - | - |
| YALI0 E24167g | Sulfite transport | -2.42 | - | - |
| YALI0 A10593g | Siderophore transmembrane transport | -2.42 | - | - |
| YALI0 C17963g | Unknown function | -2.42 | - | - |
| YALI0 E34947g | Polyphosphate catabolic process | -2.42 | - | - |
| YALI0 B16192g | Unknown function | -2.42 | - | - |
| YALI0 F11616g | Unknown function | -2.45 | - | - |
| YALI0 C04884g | Membrane | -2.45 | - | - |
| YALI0 E21307g | L-lactate dehydrogenase (cytochrome) activity | -2.46 | - | - |
| YALI0 B13134g | Iron ion transport\|plasma membrane | -2.46 | - | - |
| YALI0 C21274g | Unknown function | -2.47 | - | - |
| YALI0 F19118g | Siderophore transmembrane transport | -2.50 | - | - |
| YALI0 A00979g | Zinc ion transmembrane transport | -2.51 | - | - |
| YALI0 B21780g | Alditol:NADP+ 1-oxidoreductase activity\|cytosol | -2.53 | - | - |
| YALI0 F30569g | Cytosine transport | -2.53 | - | - |
| YALI0 E15840g | Formate dehydrogenase (NAD+) activity | -2.61 | - | - |
| YALI0 E05313g | Fungal-type vacuole membrane | -2.63 | - | - |
| YALI0 A11803g | Unknown function | -2.64 | - | - |
| YALI0 C19822g | Unknown function | -2.65 | - | - |
| YALI0 F11627g | L-cysteine catabolic process | -2.72 | - | - |
| YALI0 B21846g | Unknown function | -2.73 | - | - |
| YALI0 F20262g | Unknown function | -2.77 | - | - |
| YALI0 D04092g | Alditol:NADP+ 1-oxidoreductase activity | -2.85 | - | - |
| YALI0 E18348g | Alditol:NADP+ 1-oxidoreductase activity | -2.88 | - | - |
| YALI0 B12760g | Unknown function | -2.89 | - | - |
| YALI0 C24035g | Unknown function | -2.91 | - | - |
| YALI0 D23749g | Regulation of transcription by RNA polymerase II | -2.91 | - | - |
| YALI0 B21824g | Unknown function | -3.05 | - | - |
| YALI0 D12661g | Unknown function | -3.11 | - | - |
| YALI0 E00264g | Aldehyde dehydrogenase (NAD+) activity | -3.13 | - | - |
| YALI0 C06908g | Unknown function | -3.24 | - | - |
| YALI0 D09933g | Alternative oxidase activity | -3.41 | - | - |
| YALI0 E01562g | Unknown function | -3.44 | - | - |
| YALI0 F19536g | Transmembrane transporter activity | -3.61 | - | - |
| YALI0 A14949g | Unknown function | -3.63 | - | - |
| YALI0 D00759g | Zinc ion transmembrane transport | -3.74 | - | - |
| YALI0 C02541g | Siderophore transmembrane transport | -3.78 | - | - |
| YALI0 B16214g | L-serine catabolic process | -3.80 | - | - |
| YALI0 A08195g | Unknown function | -3.84 | - | - |
| YALI0 D11638g | Unknown function | -3.85 | - | - |
| YALI0 D00495g | Unknown function | -3.93 | - | - |
| YALI0 F25553g | Carbohydrate:proton symporter activity | -3.95 | - | - |
| YALI0 D20196g | Transmembrane transporter that exports citrate across the cell membrane | -3.97 | - | - |
| YALI0 F21659g | Zinc ion transmembrane transport | -4.25 | - | - |
| YALI0 D10021g | Unknown function | -4.34 | - | - |
| YALI0 D07304g | Iron ion transmembrane transport | -5.03 | - | - |
| YALI0 D19338g | Unknown function | -6.62 | - | - |
| YALI0 B13090g | Iron ion transport | -8.09 | - | - |
| GO:0005747 | Mitochondrial respiratory chain complex I | - | 0.0002 | - |
| GO:0005750 | Mitochondrial respiratory chain complex III | - | 0.0017 | - |
| GO:0006122 | Mitochondrial electron transport. ubiquinol to cytochrome c | - | 0.0017 | - |
| GO:0005783 | Endoplasmic reticulum | - | 0.0027 | - |
| GO:0006696 | Ergosterol biosynthetic process | - | 0.0027 | - |
| GO:0006123 | Mitochondrial electron transport. cytochrome c to oxygen | - | 0.0029 | - |
| GO:0005885 | Arp2/3 protein complex | - | 0.0364 | - |
| GO:0034314 | Arp2/3 complex-mediated actin nucleation | - | 0.0364 | - |
| GO:0016405 | CoA-ligase activity | - | 0.0496 | - |
| GO:0005730 | Nucleolus | - | 0.0000 | - |
| GO:0000293 | Ferric-chelate reductase activity | - | 0.0230 | - |
| GO:0032040 | Small-subunit processome | - | 0.0472 | - |
| GO:0006826 | Iron ion transport | - | 0.0472 | - |
| KEGG:yli01100 | Global and overview maps - metabolic pathways | - | 0.0000 | - |
| KEGG:yli00190 | Energy metabolism - oxydative phosphorilation | - | 0.0000 | - |
| KEGG:yli01110 | Global and overview maps - biosynthesis of secondary metabolites | - | 0.0000 | - |
| KEGG:yli00100 | Lipid metabolism - steroid biosynthesis | - | 0.0000 | - |
| KEGG:yli00061 | Lipid metabolism - fatty acid biosynthesis | - | 0.0014 | - |
| KEGG:yli01212 | Global and overview maps - fatty acid metabolism | - | 0.0067 | - |
| KEGG:yli03430 | Replication and repair - mismatch repair | - | 0.0000 | - |
| KEGG:yli03030 | Replication and repair - DNA replication | - | 0.0054 | - |
| KEGG:yli01230 | Global and overview maps - biosynthesis of amino acids | - | 0.0238 | - |
| KEGG:yli00630 | Carbohydrate metabolism - glyoxylate and dicarboxylate metabolism | - | 0.0238 | - |
| YALI0 C03443g | Response to hydrogen peroxide | - | - | 12.05 |
| YALI0 D17864g | Long-chain fatty acid-CoA ligase activity | - | - | 10.52 |
| YALI0 C11341g | Plasma membrane lipid binding | - | - | 11.67 |

**Supplementary Table 2.** Significant up- or down-regulated genes within a -2<logFC>2. GO:Terms, KEGG pathways (padj<0.05) and high expression genes (logCPM>10) identified through Evo-E vs. WT-E are included in this table. Up-regulation is represented in red while down-regulation in blue. Information about the corresponding gene product was obtained from NCBI database at ([NCBI](https://www.ncbi.nlm.nih.gov/)).

**ID Description/Function logFC padj logCPM**

| YALI0 F15763g | Microtubule cytoskeleton organization | 2.01 | - | - |
| --- | --- | --- | --- | --- |
| YALI0 A12199g | Unknown function | 2.02 | - | - |
| YALI0 D06864g | Calcium activated cation channel activity | 2.02 | - | - |
| YALI0 C14630g | Unknown function | 2.03 | - | - |
| YALI0 A21076g | Unknown function | 2.04 | - | - |
| YALI0 A18920g | Transmembrane transport | 2.04 | - | - |
| YALI0 E17677g | Cellular zinc ion homeostasis | 2.04 | - | - |
| YALI0 B17182g | Unknown function | 2.04 | - | - |
| YALI0 A19536g | Sphingolipid biosynthetic process | 2.04 | - | - |
| YALI0 E13552g | Histone lysine N-methyltransferase activity | 2.06 | - | - |
| YALI0 E03388g | Mitotic actomyosin contractile ring assembly | 2.06 | - | - |
| YALI0 E01408g | Phosphatidylinositol dephosphorylation | 2.07 | - | - |
| YALI0 F13805g | Unknown function | 2.07 | - | - |
| YALI0 A12177g | 2-oxoglutarate-dependent dioxygenase activity | 2.08 | - | - |
| YALI0 D17248g | Unknown function | 2.08 | - | - |
| YALI0 F13343g | Microfilament motor activity | 2.08 | - | - |
| YALI0 D00781g | Unknown function | 2.09 | - | - |
| YALI0 E14168g | Unknown function | 2.09 | - | - |
| YALI0 F00462g | Water channel activity | 2.09 | - | - |
| YALI0 E16038g | Positive regulation of cytokinesis | 2.09 | - | - |
| YALI0 B01210g | Unknown function | 2.10 | - | - |
| YALI0 A17875g | Cellular aldehyde metabolic process | 2.10 | - | - |
| YALI0 B21582g | Response to starvation | 2.12 | - | - |
| YALI0 F00990g | Unknown function | 2.13 | - | - |
| YALI0 A09559g | Unknown function | 2.15 | - | - |
| YALI0 B19228g | Regulation of cell shape | 2.15 | - | - |
| YALI0 E12309g | CAAX-box protein processing | 2.16 | - | - |
| YALI0 B10626g | Unknown function | 2.17 | - | - |
| YALI0 F24189g | Unknown function | 2.19 | - | - |
| YALI0 D26466g | Unknown function | 2.19 | - | - |
| YALI0 A14883g | Siderophore-iron transmembrane transporter activity | 2.19 | - | - |
| YALI0 E10417g | Cell wall chitin biosynthetic process | 2.20 | - | - |
| YALI0 F25883g | Unknown function | 2.21 | - | - |
| YALI0 E29491g | Unknown function | 2.21 | - | - |
| YALI0 C12496g | Unknown function | 2.22 | - | - |
| YALI0 F08701g | Unknown function | 2.22 | - | - |
| YALI0 B05874g | Unknown function | 2.23 | - | - |
| YALI0 D26015g | Mitotic cell cycle | 2.23 | - | - |
| YALI0 A00506g | Mitotic cell cycle | 2.23 | - | - |
| YALI0 E16973g | DNA-binding transcription factor activity. RNA polymerase II-specific | 2.24 | - | - |
| YALI0 A07667g | Unknown function | 2.25 | - | - |
| YALI0 F28567g | Enoyl-CoA hydratase activity | 2.25 | - | - |
| YALI0 D10703g | Unknown function | 2.26 | - | - |
| YALI0 A18425g | Unknown function | 2.26 | - | - |
| YALI0 A19250g | Unknown function | 2.26 | - | - |
| YALI0 E06831g | Peroxisome matrix targeting signal-2 binding | 2.27 | - | - |
| YALI0 F29711g | Transmembrane transporter activity | 2.27 | - | - |
| YALI0 E33561g | Unknown function | 2.28 | - | - |
| YALI0 E23185g | Long-chain fatty acyl-CoA binding | 2.28 | - | - |
| YALI0 E32263g | Unknown function | 2.30 | - | - |
| YALI0 E32065g | Ergosterol biosynthetic process | 2.30 | - | - |
| YALI0 D20900g | Unknown function | 2.31 | - | - |
| YALI0 F25487g | Acyl-CoA hydrolase activity | 2.32 | - | - |
| YALI0 F13981g | Transmembrane transporter activity | 2.32 | - | - |
| YALI0 E24673g | Cell wall chitin metabolic process | 2.35 | - | - |
| YALI0 C03377g | Ubiquitin ligase activator activity | 2.37 | - | - |
| YALI0 E07579g | Unknown function | 2.37 | - | - |
| YALI0 C12408g | Unknown function | 2.39 | - | - |
| YALI0 C08349g | Unknown function | 2.41 | - | - |
| YALI0 F25619g | Fungal-type vacuole membrane | 2.41 | - | - |
| YALI0 E28402g | Unknown function | 2.42 | - | - |
| YALI0 E29271g | DNA-binding transcription factor activity. RNA polymerase II-specific | 2.43 | - | - |
| YALI0 A19646g | Unknown function | 2.45 | - | - |
| YALI0 A09086g | Unknown function | 2.45 | - | - |
| YALI0 E09581g | Unknown function | 2.47 | - | - |
| YALI0 F18304g | Unknown function | 2.48 | - | - |
| YALI0 B12738g | Unknown function | 2.49 | - | - |
| YALI0 D11088g | cytoplasm | 2.49 | - | - |
| YALI0 A10703g | Unknown function | 2.54 | - | - |
| YALI0 E25828g | Unknown function | 2.55 | - | - |
| YALI0 F22011g | Unknown function | 2.55 | - | - |
| YALI0 B10604g | Unknown function | 2.55 | - | - |
| YALI0 E19899g | Unknown function | 2.56 | - | - |
| YALI0 B17160g | Unknown function | 2.59 | - | - |
| YALI0 C17105g | Transmembrane transporter activity | 2.59 | - | - |
| YALI0 C19239g | Unknown function | 2.61 | - | - |
| YALI0 D12232g | Unknown function | 2.61 | - | - |
| YALI0 B19206g | Regulation of cyclin-dependent protein serine/threonine kinase activity | 2.64 | - | - |
| YALI0 D17270g | Aspartic-type endopeptidase activity | 2.66 | - | - |
| YALI0 E08932g | Unknown function | 2.67 | - | - |
| YALI0 F07128g | Unknown function | 2.69 | - | - |
| YALI0 C19866g | Unknown function | 2.69 | - | - |
| YALI0 B14014g | Oxidoreductase activity | 2.70 | - | - |
| YALI0 F15961g | Unknown function | 2.72 | - | - |
| YALI0 D12100g | Transmembrane transporter activity | 2.73 | - | - |
| YALI0 A06699g | Unknown function | 2.73 | - | - |
| YALI0 F21384g | Unknown function | 2.73 | - | - |
| YALI0 C15532g | Serine-type endopeptidase activity | 2.76 | - | - |
| YALI0 F08085g | Unknown function | 2.77 | - | - |
| YALI0 A06721g | Unknown function | 2.79 | - | - |
| YALI0 E20823g | Aspartic-type endopeptidase activity | 2.79 | - | - |
| YALI0 A02475g | Unknown function | 2.79 | - | - |
| YALI0 B06028g | Unknown function | 2.81 | - | - |
| YALI0 E25443g | Unknown function | 2.83 | - | - |
| YALI0 F21428g | Unknown function | 2.83 | - | - |
| YALI0 B19866g | Unknown function | 2.87 | - | - |
| YALI0 B21142g | Fungal-type vacuole membrane | 2.91 | - | - |
| YALI0 C04389g | Unknown function | 2.91 | - | - |
| YALI0 D25916g | Unknown function | 2.91 | - | - |
| YALI0 F06556g | CoA-ligase activity | 2.96 | - | - |
| YALI0 D24277g | Unknown function | 2.98 | - | - |
| YALI0 F07040g | Iron ion transport\|plasma membrane | 2.99 | - | - |
| YALI0 D03465g | Unknown function | 3.01 | - | - |
| YALI0 C08473g | Unknown function | 3.06 | - | - |
| YALI0 C20801g | Unknown function | 3.07 | - | - |
| YALI0 E04851g | Unknown function | 3.10 | - | - |
| YALI0 B18370g | Unknown function | 3.15 | - | - |
| YALI0 A11649g | Unknown function | 3.18 | - | - |
| YALI0 D17292g | Unknown function | 3.29 | - | - |
| YALI0 F14993g | Unknown function | 3.37 | - | - |
| YALI0 F11737g | Unknown function | 3.38 | - | - |
| YALI0 C14696g | Unknown function | 3.39 | - | - |
| YALI0 D19602g | Unknown function | 3.43 | - | - |
| YALI0 F23199g | Unknown function | 3.43 | - | - |
| YALI0 D25586g | Unknown function | 3.56 | - | - |
| YALI0 C21384g | Unknown function | 3.58 | - | - |
| YALI0 E12045g | Unknown function | 3.61 | - | - |
| YALI0 A00176g | Unknown function | 3.62 | - | - |
| YALI0 B09955g | Unknown function | 3.64 | - | - |
| YALI0 E10659g | a triacylglycerol + H_2_O = a diacylglycerol + a fatty acid + H^+^ | 3.74 | - | - |
| YALI0 D01023g | Unknown function | 3.87 | - | - |
| YALI0 A16423g | DNA binding | 3.97 | - | - |
| YALI0 D00154g | Unknown function | 4.00 | - | - |
| YALI0 E06127g | Unknown function | 4.04 | - | - |
| YALI0 C22836g | Unknown function | 4.08 | - | - |
| YALI0 C23452g | Unknown function | 4.18 | - | - |
| YALI0 A16313g | Unknown function | 4.24 | - | - |
| YALI0 D13288g | Unknown function | 4.42 | - | - |
| YALI0 A17556g | Unknown function | 4.45 | - | - |
| YALI0 D09185g | Unknown function | 4.74 | - | - |
| YALI0 A19118g | Unknown function | 4.82 | - | - |
| YALI0 F10901g | Unknown function | 4.83 | - | - |
| YALI0 C15268g | Unknown function | 4.90 | - | - |
| YALI0 C19558g | mRNA binding | 5.29 | - | - |
| YALI0 F16962g | Unknown function | 5.40 | - | - |
| YALI0 F14157g | Unknown function | 5.55 | - | - |
| YALI0 F21285g | Unknown function | 7.32 | - | - |
| YALI0 D02189g | Unknown function | 8.30 | - | - |
| YALI0 D24145g | Fluoride transmembrane transport | 9.89 | - | - |
| YALI0 B21868g | Unknown function | -2.00 | - | - |
| YALI0 F18326g | Regulation of transcription by RNA polymerase II | -2.03 | - | - |
| YALI0 F20262g | Unknown function | -2.03 | - | - |
| YALI0 C07821g | Unknown function | -2.04 | - | - |
| YALI0 D15664g | DNA binding | -2.07 | - | - |
| YALI0 E02024g | Unknown function | -2.08 | - | - |
| YALI0 D24189g | ATPase-coupled transmembrane transporter activity | -2.08 | - | - |
| YALI0 E03872g | Unknown function | -2.09 | - | - |
| YALI0 D12661g | Unknown function | -2.12 | - | - |
| YALI0 B20284g | Regulation of transcription by RNA polymerase II | -2.18 | - | - |
| YALI0 A11803g | Unknown function | -2.21 | - | - |
| YALI0 E24057g | Unknown function | -2.25 | - | - |
| YALI0 B09625g | Proline catabolic process to glutamate | -2.27 | - | - |
| YALI0 A19866g | Unknown function | -2.33 | - | - |
| YALI0 D00132g | Transmembrane transport | -2.43 | - | - |
| YALI0 B21846g | Unknown function | -2.43 | - | - |
| YALI0 B01364g | Unknown function | -2.44 | - | - |
| YALI0 A08195g | Unknown function | -2.44 | - | - |
| YALI0 E00110g | Phospholipid catabolic process | -2.44 | - | - |
| YALI0 C09031g | Unknown function | -2.45 | - | - |
| YALI0 F05984g | Transmembrane transporter activity | -2.47 | - | - |
| YALI0 F19646g | Unknown function | -2.51 | - | - |
| YALI0 E24167g | Sulfite transmembrane transporter activity | -2.52 | - | - |
| YALI0 E00264g | Aldehyde dehydrogenase (NAD+) activity | -2.53 | - | - |
| YALI0 F25685g | Unknown function | -2.54 | - | - |
| YALI0 D07304g | Iron ion transmembrane transport | -2.56 | - | - |
| YALI0 B21780g | Alditol:NADP+ 1-oxidoreductase activity | -2.58 | - | - |
| YALI0 A15906g | Alditol:NADP+ 1-oxidoreductase activity | -2.59 | - | - |
| YALI0 B16214g | L-serine catabolic process | -2.60 | - | - |
| YALI0 D00495g | Unknown function | -2.61 | - | - |
| YALI0 E22506g | L-amino acid transmembrane transporter activity | -2.62 | - | - |
| YALI0 B17776g | Transmembrane transporter activity | -2.65 | - | - |
| YALI0 F11627g | L-cysteine catabolic process | -2.70 | - | - |
| YALI0 B16192g | Unknown function | -2.76 | - | - |
| YALI0 B13112g | Iron ion transport | -2.76 | - | - |
| YALI0 E34749g | Hydrogen peroxide catabolic process | -2.81 | - | - |
| YALI0 C04884g | Membrane | -2.83 | - | - |
| YALI0 E12507g | Unknown function | -3.05 | - | - |
| YALI0 B21824g | Unknown function | -3.29 | - | - |
| YALI0 D04092g | Alditol:NADP+ 1-oxidoreductase activity | -3.45 | - | - |
| YALI0 F25553g | Carbohydrate transport | -3.76 | - | - |
| YALI0 B13090g | Iron ion transport | -4.74 | - | - |
| YALI0 C02541g | Siderophore transmembrane transport | -5.36 | - | - |
| GO:0006696 | Ergosterol biosynthetic process | - | 0.0005 | - |
| GO:0051015 | Actin filament binding | - | 0.0013 | - |
| GO:0005783 | Endoplasmic reticulum | - | 0.0013 | - |
| GO:0000070 | Mitotic sister chromatid segregation | - | 0.0187 | - |
| GO:0005730 | Nucleolus | - | 0.0000 | - |
| GO:0032040 | Small-subunit processome | - | 0.0000 | - |
| GO:0003735 | Structural constituent of ribosome | - | 0.0000 | - |
| GO:0000462 | Maturation of SSU-rRNA from tricistronic rRNA transcript (SSU-rRNA. 5.8S rRNA. LSU-rRNA) | - | 0.0000 | - |
| GO:0022625 | Cytosolic large ribosomal subunit | - | 0.0000 | - |
| GO:0042273 | Ribosomal large subunit biogenesis | - | 0.0000 | - |
| GO:0005762 | Mitochondrial large ribosomal subunit | - | 0.0000 | - |
| GO:0003723 | RNA binding | - | 0.0001 | - |
| GO:0030687 | Preribosome. Large subunit precursor | - | 0.0001 | - |
| GO:0000470 | Maturation of LSU-rRNA | - | 0.0002 | - |
| GO:0006364 | rRNA processing | - | 0.0028 | - |
| GO:0006412 | Translation | - | 0.0036 | - |
| GO:0030686 | 90S preribosome | - | 0.0047 | - |
| GO:0031305 | Integral component of mitochondrial inner membrane | - | 0.0047 | - |
| GO:0000460 | Maturation of 5.8S rRNA | - | 0.0070 | - |
| GO:0005763 | Mitochondrial small ribosomal subunit | - | 0.0089 | - |
| GO:0000027 | Ribosomal large subunit assembly | - | 0.0375 | - |
| GO:0030150 | Protein import into mitochondrial matrix | - | 0.0380 | - |
| GO:0019843 | rRNA binding | - | 0.0386 | - |
| GO:0030515 | snoRNA binding | - | 0.0389 | - |
| GO:0030688 | Preribosome. Small subunit precursor | - | 0.0457 | - |
| GO:0003899 | DNA-directed 5'-3' RNA polymerase activity | - | 0.0457 | - |
| GO:0007005 | Mitochondrion organization | - | 0.0457 | - |
| KEGG:yli04111 | Cell cycle | - | 0.0000 | - |
| KEGG:yli00100 | Steroid biosynthesis | - | 0.0000 | - |
| KEGG:yli04146 | Peroxisome | - | 0.0000 | - |
| KEGG:yli01100 | Metabolic pathways | - | 0.0000 | - |
| KEGG:yli04113 | Meiosis | - | 0.0102 | - |
| KEGG:yli00510 | N-Glycan biosynthesis | - | 0.0109 | - |
| KEGG:yli00592 | α-Linolenic acid metabolism | - | 0.0325 | - |
| KEGG:yli04392 | Hippo signaling pathway | - | 0.0325 | - |
| KEGG:yli01040 | Biosynthesis of unsaturated fatty acids | - | 0.0424 | - |
| KEGG:yli00900 | Terpenoid backbone biosynthesis | - | 0.0424 | - |
| KEGG:yli00562 | Inositol phosphate metabolism | - | 0.0424 | - |
| KEGG:yli01110 | Biosynthesis of secondary metabolites | - | 0.0424 | - |
| KEGG:yli00061 | Fatty acid biosynthesis | - | 0.0499 | - |
| KEGG:yli03010 | Ribosome | - | 0.0000 | - |
| KEGG:yli03008 | Ribosome biogenesis in eukaryotes | - | 0.0000 | - |
| KEGG:yli03020 | RNA polymerase | - | 0.0000 | - |
| YALI0 C03443g | Response to hydrogen peroxide | - | - | 11.75 |
| YALI0 E16016g | Lipid droplet | - | - | 10.46 |
| YALI0 C23859g | Peroxisome | - | - | 10.55 |
| YALI0 A19536g | Sphingolipid biosynthetic process | - | - | 10.23 |
| YALI0 A01023g | Cellular calcium ion homeostasis | - | - | 10.68 |
| YALI0 D06688g | Iron ion transmembrane transport | - | - | 10.60 |
| YALI0 B19250g | Siderophore-iron transmembrane transporter activity | - | - | 12.00 |
| YALI0 B19800g | Amino acid transmembrane transport | - | - | 11.93 |

**Supplementary Table 3.** Significant up- or down-regulated genes within a -2<logFC>2. GO:Terms, KEGG pathways (padj<0.05) and high expression genes (logCPM>10) identified through Evo-B vs. Evo-E are included in this table. Up-regulation is represented in red while down-regulation in blue. Information about the corresponding gene product was obtained from NCBI database at ([NCBI](https://www.ncbi.nlm.nih.gov/)).

**ID Description/Function logFC padj logCPM**

| YALI0 C12496g | Unknown function | 2.02 | - | - |
| --- | --- | --- | --- | --- |
| YALI0 D11880g | Protein localization to bud neck | 2.02 | - | - |
| YALI0 E33825g | Unknown function | 2.02 | - | - |
| YALI0 E14168g | Unknown function | 2.03 | - | - |
| YALI0 A09086g | Unknown function | 2.04 | - | - |
| YALI0 B19206g | Regulation of cyclin-dependent protein serine/threonine kinase activity | 2.07 | - | - |
| YALI0 B12738g | Unknown function | 2.08 | - | - |
| YALI0 F18282g | Unknown function | 2.10 | - | - |
| YALI0 D11088g | cytoplasm | 2.12 | - | - |
| YALI0 F00462g | Water channel activity | 2.14 | - | - |
| YALI0 D12232g | Unknown function | 2.15 | - | - |
| YALI0 F13343g | Microfilament motor activity | 2.15 | - | - |
| YALI0 E06809g | Cellular bud neck | 2.18 | - | - |
| YALI0 B06413g | Oxidoreductase activity | 2.20 | - | - |
| YALI0 D15400g | Biotin biosynthetic process | 2.21 | - | - |
| YALI0 A00506g | Mitotic cell cycle | 2.23 | - | - |
| YALI0 E31108g | Unknown function | 2.26 | - | - |
| YALI0 F09581g | DNA binding | 2.32 | - | - |
| YALI0 A16445g | Primary amine oxidase activity | 2.34 | - | - |
| YALI0 B21142g | Fungal-type vacuole membrane | 2.37 | - | - |
| YALI0 A00176g | Unknown function | 2.50 | - | - |
| YALI0 A15125g | Unknown function | 2.58 | - | - |
| YALI0 E08932g | Unknown function | 2.62 | - | - |
| YALI0 F07128g | Unknown function | 2.63 | - | - |
| YALI0 B09955g | Unknown function | 2.64 | - | - |
| YALI0 B23496g | Unknown function | 2.64 | - | - |
| YALI0 D09185g | Unknown function | 2.71 | - | - |
| YALI0 A21373g | Unknown function | 2.81 | - | - |
| YALI0 C04411g | Cellular manganese ion homeostasis | 2.91 | - | - |
| YALI0 D00154g | Unknown function | 2.94 | - | - |
| YALI0 F16962g | Unknown function | 3.25 | - | - |
| YALI0 A21351g | Unknown function | 3.35 | - | - |
| YALI0 C23452g | Unknown function | 3.78 | - | - |
| YALI0 F21285g | Unknown function | 5.22 | - | - |
| YALI0 F28369g | Transmembrane transporter activity | 5.26 | - | - |
| YALI0 F11825g | Iron ion transport | -2.03 | - | - |
| YALI0 F30437g | Unknown function | -2.15 | - | - |
| YALI0 C09031g | Unknown function | -2.15 | - | - |
| YALI0 C06908g | Unknown function | -2.28 | - | - |
| YALI0 B23452g | Unknown function | -2.37 | - | - |
| YALI0 C01001g | Unknown function | -2.37 | - | - |
| YALI0 D15422g | Amino acid transmembrane transport | -2.48 | - | - |
| YALI0 B17776g | Transmembrane transporter activity | -2.82 | - | - |
| YALI0 E05819g | rRNA processing | -2.91 | - | - |
| YALI0 A19866g | Unknown function | -3.11 | - | - |
| YALI0 C02541g | Siderophore-iron transmembrane transporter activity | -3.30 | - | - |
| YALI0 B19492g | Amino acid transmembrane transport | -3.87 | - | - |
| YALI0 B13112g | Iron ion transport | -3.90 | - | - |
| YALI0 E29271g | DNA-binding transcription factor activity. RNA polymerase II-specific | -3.96 | - | - |
| YALI0 C15004g | Unknown function | -4.49 | - | - |
| YALI0 C20060g | Unknown function | -7.35 | - | - |
| GO:0005874 | Microtubule | - | 0.0003 | - |
| GO:0005778 | Peroxisomal membrane | - | 0.0005 | - |
| GO:0003777 | Microtubule motor activity | - | 0.0033 | - |
| GO:0005871 | Kinesin complex | - | 0.0033 | - |
| GO:0000226 | Microtubule cytoskeleton organization | - | 0.0033 | - |
| GO:0007076 | Mitotic chromosome condensation | - | 0.0041 | - |
| GO:0008017 | Microtubule binding | - | 0.0041 | - |
| GO:0007018 | Microtubule-based movement | - | 0.0042 | - |
| GO:0005886 | Plasma membrane | - | 0.0178 | - |
| GO:0006086 | Acetyl-CoA biosynthetic process from pyruvate | - | 0.0282 | - |
| GO:0016020 | Membrane | - | 0.0405 | - |
| KEGG:yli04146 | Peroxisome | - | 0.0000 | - |
| KEGG:yli04111 | Cell cycle | - | 0.0000 | - |
| KEGG:yli01100 | Metabolic pathways | - | 0.0000 | - |
| KEGG:yli01110 | Biosynthesis of secondary metabolites | - | 0.0000 | - |
| KEGG:yli01230 | Biosynthesis of amino acids | - | 0.0005 | - |
| KEGG:yli01210 | 2-Oxocarboxylic acid metabolism | - | 0.0011 | - |
| KEGG:yli00270 | Cysteine and methionine metabolism | - | 0.0020 | - |
| KEGG:yli00650 | Butanoate metabolism | - | 0.0025 | - |
| KEGG:yli00280 | Valine. leucine and isoleucine degradation | - | 0.0115 | - |
| KEGG:yli00670 | One carbon pool by folate | - | 0.0128 | - |
| KEGG:yli04392 | Hippo signaling pathway | - | 0.0143 | - |
| KEGG:yli01240 | Biosynthesis of cofactors | - | 0.0276 | - |
| KEGG:yli00100 | Steroid biosynthesis | - | 0.0276 | - |
| KEGG:yli00290 | Valine. Leucine and isoleucine biosynthesis | - | 0.0312 | - |
| KEGG:yli00400 | Phenylalanine. Tyrosine and tryptophan biosynthesis | - | 0.0355 | - |
| KEGG:yli00260 | Glycine. Serine and threonine metabolism | - | 0.0368 | - |
| KEGG:yli00770 | Pantothenate and CoA biosynthesis | - | 0.0410 | - |
| KEGG:yli00592 | α-Linolenic acid metabolism | - | 0.0423 | - |
| YALI0 C23859g | Peroxisome | - | - | 10.00 |
| YALI0 B22066g | P-type proton-exporting transporter activity | - | - | 11.29 |
| YALI0 B19250g | Siderophore-iron transmembrane transporter activity | - | - | 12.00 |
| YALI0 D06688g | Iron ion transmembrane transport | - | - | 10.60 |
| YALI0 F15411g | Zinc ion transmembrane transport | - | - | 11.68 |
| YALI0 D20526g | Lipid binding | - | - | 13.31 |

**Supplementary Table 4.** Significant genes within a -2<logFC>2. GO:Terms. KEGG pathways (padj<0.05) and high expression genes (logCPM>10) identified through WT-B vs. WT-E. Upregulation has been represented in red and downregulation in blue. Information about the corresponding gene product was obtained from NCBI database at ([NCBI](https://www.ncbi.nlm.nih.gov/))

**ID Description/Function logFC padj logCPM**

| YALI0 D01738g | Unknown function | 2.05 | - | - |
| --- | --- | --- | --- | --- |
| YALI0 C09009g | Unknown function | 2.07 | - | - |
| YALI0 A15125g | Unknown function | 2.07 | - | - |
| YALI0 F16225g | Acetate transmembrane transporter activity | 2.12 | - | - |
| YALI0 A20812g | Unknown function | 2.15 | - | - |
| YALI0 F01650g | Oxidoreductase activity | 2.16 | - | - |
| YALI0 E32901g | Carboxylic acid transmembrane transporter activity | 2.20 | - | - |
| YALI0 F05984g | Transmembrane transporter activity | 2.20 | - | - |
| YALI0 E31108g | Unknown function | 2.24 | - | - |
| YALI0 E27291g | Acetate transmembrane transporter activity | 2.31 | - | - |
| YALI0 F21109g | Transmembrane transporter activity | 2.32 | - | - |
| YALI0 E34749g | Response to hydrogen peroxide | 2.35 | - | - |
| YALI0 E31031g | Unknown function | 2.67 | - | - |
| YALI0 A16445g | Primary amine oxidase activity | 2.67 | - | - |
| YALI0 E30789g | Regulation of transcription by RNA polymerase II | 2.67 | - | - |
| YALI0 E20691g | Glycerone kinase activity | 2.73 | - | - |
| YALI0 D00132g | Transmembrane transporter activity | 2.77 | - | - |
| YALI0 F00462g | Water channel activity | 2.92 | - | - |
| YALI0 D00363g | Carbohydrate transport | 2.94 | - | - |
| YALI0 C14696g | Unknown function | 3.04 | - | - |
| YALI0 A21373g | Unknown function | 3.06 | - | - |
| YALI0 F21923g | Regulation of transcription by RNA polymerase II | 3.17 | - | - |
| YALI0 D25630g | Alcohol dehydrogenase (NAD+) activity | 3.23 | - | - |
| YALI0 F20504g | Unknown function | 3.57 | - | - |
| YALI0 D12100g | Transmembrane transporter activity | 4.04 | - | - |
| YALI0 E20427g | Carbohydrate transport | 4.85 | - | - |
| YALI0 D09933g | Alternative respiration | -2.02 | - | - |
| YALI0 F07040g | Iron ion transport | -2.03 | - | - |
| YALI0 E11627g | Pheromone-dependent signal transduction involved in conjugation with cellular fusion | -2.05 | - | - |
| YALI0 D01375g | Unknown function | -2.08 | - | - |
| YALI0 F05390g | Unknown function | -2.08 | - | - |
| YALI0 B17776g | Transmembrane transporter activity | -2.09 | - | - |
| YALI0 F01386g | Iron ion transport | -2.09 | - | - |
| YALI0 E18766g | Protein localization to plasma membrane | -2.10 | - | - |
| YALI0 C10274g | Unknown function | -2.12 | - | - |
| YALI0 E11649g | Unknown function | -2.14 | - | - |
| YALI0 E27984g | Unknown function | -2.15 | - | - |
| YALI0 D25916g | Unknown function | -2.19 | - | - |
| YALI0 A09196g | Unknown function | -2.19 | - | - |
| YALI0 F30437g | Unknown function | -2.21 | - | - |
| YALI0 D26466g | Unknown function | -2.22 | - | - |
| YALI0 E25982g | Unknown function | -2.22 | - | - |
| YALI0 B14685g | Unknown function | -2.23 | - | - |
| YALI0 F15411g | Zinc ion transmembrane transport | -2.30 | - | - |
| YALI0 B09955g | Unknown function | -2.34 | - | - |
| YALI0 D23749g | Regulation of transcription by RNA polymerase II | -2.35 | - | - |
| YALI0 E04620g | Ubiquitin conjugating enzyme activity | -2.37 | - | - |
| YALI0 D09185g | Unknown function | -2.41 | - | - |
| YALI0 D13200g | Unknown function | -2.41 | - | - |
| YALI0 F19118g | Siderophore-iron transmembrane transporter activity | -2.42 | - | - |
| YALI0 C17875g | Unknown function | -2.43 | - | - |
| YALI0 D19602g | Unknown function | -2.43 | - | - |
| YALI0 B00660g | Regulation of transcription by RNA polymerase II | -2.47 | - | - |
| YALI0 A14883g | Siderophore-iron transmembrane transporter activity | -2.49 | - | - |
| YALI0 B02948g | Unknown function | -2.50 | - | - |
| YALI0 B15488g | Fungal-type vacuole membrane | -2.50 | - | - |
| YALI0 C22924g | Unknown function | -2.53 | - | - |
| YALI0 C15532g | Serine-type endopeptidase activity | -2.54 | - | - |
| YALI0 C17391g | Copper chaperone activity | -2.56 | - | - |
| YALI0 E08096g | Iron ion transport | -2.62 | - | - |
| YALI0 B01848g | Unknown function | -2.63 | - | - |
| YALI0 D17644g | Unknown function | -2.67 | - | - |
| YALI0 D27214g | Unknown function | -2.68 | - | - |
| YALI0 E11693g | DNA-binding transcription activator activity. RNA polymerase II-specific | -2.69 | - | - |
| YALI0 E00704g | Unknown function | -2.71 | - | - |
| YALI0 C03586g | Unknown function | -2.74 | - | - |
| YALI0 E11473g | Potassium ion transmembrane transporter activity | -2.77 | - | - |
| YALI0 E08074g | Deadenylation-dependent decapping of nuclear-transcribed mRNA | -2.77 | - | - |
| YALI0 A09383g | Transmembrane transporter activity | -2.85 | - | - |
| YALI0 C01001g | Unknown function | -2.86 | - | - |
| YALI0 B18298g | Copper chaperone activity | -2.93 | - | - |
| YALI0 D17270g | Aspartic-type endopeptidase activity | -2.94 | - | - |
| YALI0 C08473g | Unknown function | -2.94 | - | - |
| YALI0 D17292g | Unknown function | -2.96 | - | - |
| YALI0 E23859g | Phosphate ion transmembrane transport | -2.98 | - | - |
| YALI0 A19316g | Unknown function | -2.98 | - | - |
| YALI0 D26081g | Iron ion transport | -2.99 | - | - |
| YALI0 F01320g | Unknown function | -3.01 | - | - |
| YALI0 A19866g | Unknown function | -3.02 | - | - |
| YALI0 B10846g | Iron ion transport | -3.03 | - | - |
| YALI0 B12760g | Unknown function | -3.03 | - | - |
| YALI0 B19228g | Regulation of cell shape | -3.09 | - | - |
| YALI0 E19899g | Unknown function | -3.09 | - | - |
| YALI0 C15268g | Unknown function | -3.10 | - | - |
| YALI0 B23452g | Unknown function | -3.36 | - | - |
| YALI0 F21384g | Unknown function | -3.49 | - | - |
| YALI0 C11781g | Unknown function | -3.56 | - | - |
| YALI0 D00759g | Zinc ion transmembrane transport | -3.70 | - | - |
| YALI0 C24035g | Unknown function | -3.70 | - | - |
| YALI0 C06908g | Unknown function | -3.87 | - | - |
| YALI0 C04389g | Unknown function | -3.95 | - | - |
| YALI0 D15422g | Amino acid transmembrane transport | -3.98 | - | - |
| YALI0 A09471g | Unknown function | -4.00 | - | - |
| YALI0 D17248g | Unknown function | -4.25 | - | - |
| YALI0 F10901g | Unknown function | -4.55 | - | - |
| YALI0 E08387g | Unknown function | -4.56 | - | - |
| YALI0 C19558g | mRNA binding | -4.89 | - | - |
| YALI0 C15004g | Unknown function | -5.62 | - | - |
| YALI0 B02024g | Unknown function | -6.33 | - | - |
| YALI0 E29271g | DNA-binding transcription factor activity. RNA polymerase II-specific | -6.70 | - | - |
| YALI0 C15807g | Urea transmembrane transporter activity | -7.51 | - | - |
| GO:0022625 | Cytosolic large ribosomal subunit | - | 0.0000 | - |
| GO:0005747 | Mitochondrial respiratory chain complex I | - | 0.0000 | - |
| GO:0003735 | Structural constituent of ribosome | - | 0.0000 | - |
| GO:0022627 | Cytosolic small ribosomal subunit | - | 0.0000 | - |
| GO:0071577 | Zinc ion transmembrane transport | - | 0.0027 | - |
| GO:0000293 | Ferric-chelate reductase activity | - | 0.0027 | - |
| GO:0005385 | Zinc ion transmembrane transporter activity | - | 0.0039 | - |
| GO:0015677 | Copper ion import | - | 0.042 | - |
| KEGG:yli03010 | Ribosome | - | 0.000 | - |
| KEGG:yli00190 | Oxidative phosphorylation | - | 0.000 | - |
| KEGG:yli01100 | Metabolic pathways | - | 0.000 | - |
| KEGG:yli01110 | Biosynthesis of secondary metabolites | - | 0.000 | - |
| KEGG:yli01240 | Biosynthesis of cofactors | - | 0.001 | - |
| KEGG:yli01230 | Biosynthesis of amino acids | - | 0.001 | - |
| KEGG:yli01210 | 2-Oxocarboxylic acid metabolism | - | 0.013 | - |
| KEGG:yli00010 | Glycolysis / Gluconeogenesis | - | 0.042 | - |
| KEGG:yli01232 | Nucleotide metabolism | - | 0.0207 | - |
| KEGG:yli00230 | Purine metabolism | - | 0.0207 | - |
| KEGG:yli00240 | Pyrimidine metabolism | - | 0.0208 | - |
| KEGG:yli00440 | Phosphonate and phosphinate metabolism | - | 0.0418 | - |
| KEGG:yli03440 | Homologous recombination | - | 0.0418 | - |
| KEGG:yli00564 | Glycerophospholipid metabolism | - | 0.0418 | - |
| KEGG:yli03030 | DNA replication | - | 0.0418 | - |
| YALI0 C20295g | Copper ion transmembrane transporter activity | - | - | 10.07 |
| YALI0 F15411g | Zinc ion transmembrane transport | - | - | 11.13 |
